# An effective COVID-19 vaccine hesitancy intervention focused on the relative risks of vaccination and infection

**DOI:** 10.1038/s41598-024-57841-1

**Published:** 2024-03-28

**Authors:** Cameron O’Neill Byerley, Dru Horne, Mina Gong, Stacy Musgrave, Laura A. Valaas, Brian Rickard, Hyunkyoung Yoon, Min Sook Park, Alison Mirin, Surani Joshua, Heather Lavender, Sukjin You

**Affiliations:** 1 Department of Mathematics, Science, and Social Studies Education, University of Georgia, Athens, 30606 USA; 2 Department of Mathematics and Statistics, Cal Poly Pomona, Pomona, 91768 USA; 3 Department of Dermatology, University of Washington, Seattle, 98195 USA; 4 Department of Mathematical Sciences, University of Arkansas, Fayetteville, 72701 USA; 5 Department of Information Studies, University of Wisconsin-Milwaukee, Milwaukee, 53201 USA; 6 Department of Mathematics, University of Arizona, Tucson, 85721 USA; 7 Yavapai Elementary School, Scottsdale, 85257 USA

**Keywords:** Vaccines, Patient education, Epidemiology

## Abstract

We designed the Relative Risk Tool (RRT) to help people assess the relative risks associated with COVID-19 vaccination and infection. In May 2022 (*N* = 400) and November 2022 (*N* = 615), U.S. residents participated in a survey that included questions about the risks of vaccination and infection. In both cohorts, we found an association between relative risk perception and vaccine hesitancy. Participants in the May cohort were randomly assigned an intervention: to see information from the RRT or the Centers for Disease Control and Prevention (CDC). After the intervention, participants answered the same questions about risk perception and vaccination intent again. The RRT was more effective than the CDC at changing risk perception and increasing vaccination intent. In November, the survey structure was the same, but the RRT was the only intervention included, and we confirmed that the RRT was effective at changing opinions in this new sample. Importantly, the RRT provided accurate information about the risks of serious adverse outcomes to vaccination and still increased vaccination intent. Our work suggests that the RRT helps people assess relative risk, which can in turn help empower them to make informed decisions and ultimately reduce vaccine hesitancy.

## Introduction

COVID-19 vaccine hesitancy is a major global issue^1^, and it is important to develop research-based messaging about vaccination that is broadly understandable and supports decision making^2^. *Vaccine hesitancy* has been defined in multiple ways, including a “state of indecisiveness regarding a vaccination decision” as well as “delay in acceptance or refusal despite availability of vaccines”^3^. We consider vaccine hesitancy to be reluctance, refusal, or indecision regarding future vaccinations, but consider current vaccination status to be related to vaccine hesitancy. As of May 2023, 70.0% of the world’s population had received at least one dose of a COVID-19 vaccine^4,5^, and 17.0% of the U.S. population had received recommended boosters^6^.

People are still making decisions about vaccination^7^ and, thus, there is a need for accurate and trustworthy communication about COVID-19 vaccines^8^. Strategies for addressing vaccine hesitancy have been met with mixed success^9–14^, and effective interventions require addressing the various drivers of vaccine hesitancy with multifaceted strategies^9,15,16^. Reasons for vaccine hesitancy include concern about the risks associated with vaccination, lack of concern for the risk due to the disease, ease of access to vaccination, and mistrust in government and health institutions^8,17–24^. While reasons for vaccine hesitancy vary widely^25–27^, concern about safety and efficacy are significant reasons across populations. For example, the belief “COVID-19 vaccines are not safe” had the highest odds ratio in a statistical model that predicted vaccine hesitancy^28^. Additionally, all 14 studies in a literature review on vaccine hesitancy found that concerns about safety were associated with hesitancy^29^. In order to address people’s concerns about risks due to vaccination, we focused our research on risk perception and risk communication.

### The relative risk tool

**Figure 1:**
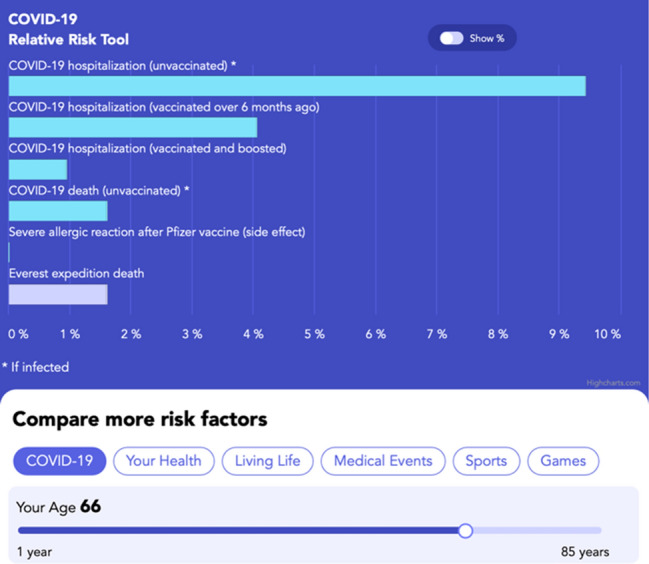
The Relative Risk Tool (https://www.covidtaser.com/relativerisk) is an interactive web app that allows users to compare the risks associated with various scenarios and COVID-19 infection after inputting their age. For example, 1.6% of mountaineers who climb above base camp at Mount Everest die, which is similar to the risk of death for an unvaccinated 66-year-old infected with COVID-19.

Based on our models of citizens’ mathematical thinking from prior mathematics education research and interviews conducted about COVID-19^30–34^, we created the RRT to help citizens compare the risks associated with COVID-19 infection with risks associated with more familiar situations such as driving, pregnancy, sports, and other events^35^. We designed, tested, and updated the Relative Risk Tool from August 2020 to May 2022 to help people understand the risks associated with COVID-19 vaccination and infection and the efficacy of vaccines^35^. Note, we will use the phrase “risk of vaccination” throughout this paper to mean “risks associated with vaccination” and likewise for infection. We were inspired to create the RRT based on research we conducted in April and May 2020 on people’s understanding of risk associated with COVID-19 infection^36^. The RRT (Fig. 1) uses bar graphs to convey that infection is more likely than vaccination to cause hospitalization and death for all ages modeled, that vaccination reduces the risks due to infection substantially, and that unvaccinated adults face more risk from infection than people are typically willing to accept as a part of daily life. The RRT incorporates an epidemiological model^37^ that allows participants to move a slider to find the average risk of hospitalization and death if an average person of a particular age was infected with COVID-19. The RRT shows both relative risk and absolute risk due to infection and vaccination.

The design of the RRT was informed by research in mathematics education and risk communication conducted before and during the pandemic^23,38–44^, with the primary aim of making the RRT understandable to most people^35,36,45,46^. The RRT provides a potential template for future risk communication tools that can be used to accurately present both absolute and relative risks to the general public using direct comparisons to related risks. Communicating risks for decision making is more difficult than simply telling people probabilities^42,47–49^ because percentage and probability are difficult concepts to learn, and people hold widely different understandings of the meaning of a particular percentage^30,31,34,50–54^. The thinking needed to accurately compare risks is more sophisticated than what is normally developed in K-12 education^55–60^. Experts often quantify risk using percentages^41,61^, and the public often uses non-numerical heuristics to estimate risk and to make decisions^62–67^. Many people unconsciously use the availability heuristic^68,69^, wherein how easily an event comes to someone’s mind influences their view of how likely that event is to occur. For example, people can readily imagine driving trips and car accidents to develop a sense of the riskiness of driving. We designed the RRT to help people make sense of the percent estimates of risk by relating COVID-19 risks to other more familiar risks like driving^70^.

The presentation format of numerical risks impacts decision making^44^, so it was critical for us to study how people understood the RRT and improve it using an iterative design-based research method. As part of the design process, we analyzed how citizens learned from the RRT using various methods such as interviewing people while using the RRT^46^, conducting online pilot surveys^45^, and thematically categorizing comments on a New York Times article which mentioned the RRT^71^ (see “Methods” for details). We also updated the RRT in response to requests for more targeted information. For example, we included the risk of myocarditis due to infection and the risk of myocarditis due to vaccination^72,73^. The version of the RRT tested in this paper was consistent with the majority of Patient Decision Aid standards with a focus on “presenting probabilities of outcomes in an unbiased and understandable way” to help people understand positive and negative potential outcomes of vaccination^74^. As recommended by the Patient Decision Aid Standards, the RRT also used plain language, disclosed funding, cited peer-reviewed sources of information, and had a systematic development process.

### Overview of study

**Figure 2:**
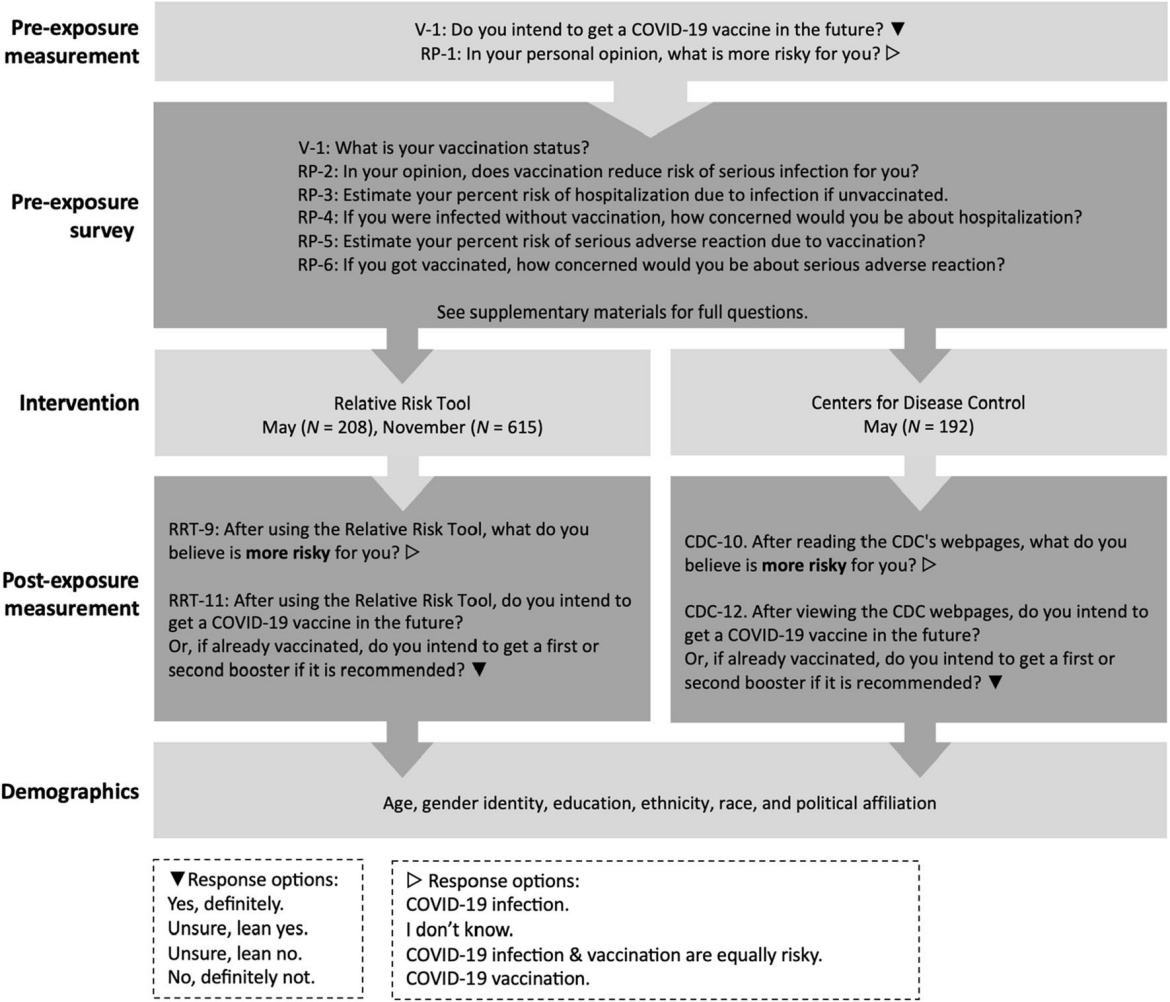
Design of study. All participants were given questions pre-exposure to the intervention about their vaccination status, intention to be vaccinated, and questions about risk. Post exposure participants were asked about vaccination intent, relative risk, and demographics. The questions are abbreviated and labeled in this figure (V-1, RP-1, etc.) with full questions available in supplementary materials and further description in “Methods” section.

We tested the 2022 version of the RRT (current version available at http://www.covidtaser.com) in this study. This paper (1) models the relationship between risk perception and COVID-19 vaccination status and intent. This paper, then, (2) evaluates if the RRT intervention, designed specifically to educate about relative risks associated with COVID-19 infection and vaccination, changes participants’ risk perception or intent to be vaccinated. Based on our interviews and prior regression models^8^, we hypothesized that beliefs about the relative risks of vaccination and infection would be correlated with vaccination status and future vaccination intent. Therefore, we also hypothesized that education about relative risks of vaccination and infection would help people resolve indecision about vaccination and increase their willingness to accept future vaccines. To understand the impact of the RRT, we conducted an online pre-post exposure study in May and November 2022 with U.S. adult residents, outlined in Fig. 2. In May, half of the participants saw information from the RRT, and the other half constituted a control group that was randomly assigned to see similar information about safety and efficacy of the COVID-19 vaccine from the CDC website (see supplementary materials). All study participants answered demographic questions, questions about risk (inspired by our earlier interviews), and questions from two large studies on vaccination intent^8,75^. We assessed the causal impact of information about safety and efficacy on two dimensions: (1) Belief about the relative risk of COVID-19 vaccination and infection and (2) Intent to receive a COVID-19 vaccine in the future. By May 2022, both the Pfizer and Moderna vaccines were fully approved by the U.S. Food and Drug Administration and freely available to all adults. Because the May survey demonstrated larger effects with the RRT than the CDC, and because we wanted to be able to analyze effects of the RRT in subgroups, we opted to not assign participants to view the CDC website in November.

## Results

### Sample characteristics

We surveyed U.S. residents using Prime Panels, which samples from a population similar to the U.S. population^76^. The surveys were conducted on May 24, 2022 (*N* = 400) and November 11, 2022 (*N* = 615). Supplementary Table 1 provides information on the sample demographics with the self-reported age, education level, gender, race, ethnicity, political affiliation, COVID-19 infection history, and COVID-19 vaccination status. There are no statistically significant differences in participants’ demographics between the May and November samples.

### U.S. residents’ risk perceptions

Table 1 displays results of pre-intervention questions about risk perception.

**Table 1:** Responses to questions about COVID-19 infection risk and COVID-19 vaccination risk. Supplementary Figs. 1, 2, and 3 have participants’ reasons for relative risk assessments.

	May 2022 (%) *N* = 400	Nov 2022 (%) *N* = 615
**Vaccination status**		
Vaccinated and boosted	44.5	51.9
Vaccinated without booster	20.0	18.9
Partially vaccinated (1 shot Pfizer or Moderna)	4.5	3.7
Unvaccinated	31.0	25.5
**What is more risky for you?**		
COVID-19 infection	48.75	54.5
COVID-19 vaccination	12.75	15.4
COVID-19 infection and vaccination are equally risky	25.75	18.7
I don’t know	12.75	11.4
**Does the COVID-19 vaccine reduce risk of severe COVID-19 infection outcomes for you?**		
Yes	57.0	60.7
No	23.0	23.7
I don’t know	20.0	15.6
**Approximately how many U.S. citizens have died primarily because of COVID-19 vaccination?**		
No one has died due to COVID-19 vaccination	22.5	25.5
Fewer than 50 people	19.5	20.5
Between 50 and 500 people	20.75	20.2
Between 500 and 50,000 people	18.25	16.8
Between 50,000 and 500,000 people	10.5	9.4
Over 500,000 people have died due to vaccination	8.5	7.6
**Approximately how many U.S. citizens have died primarily because of COVID-19 infection?**		
No one has died due to COVID-19 infection	6.0	6.7
Fewer than 50 people	6.5	8.3
Between 50 and 500 people	9.75	13.7
Between 500 and 50,000 people	18.5	18.2
Between 50,000 and 500,000 people	20.5	17.2
Over 500,000 people have died due to infection	38.75	35.9

**Figure 3:**
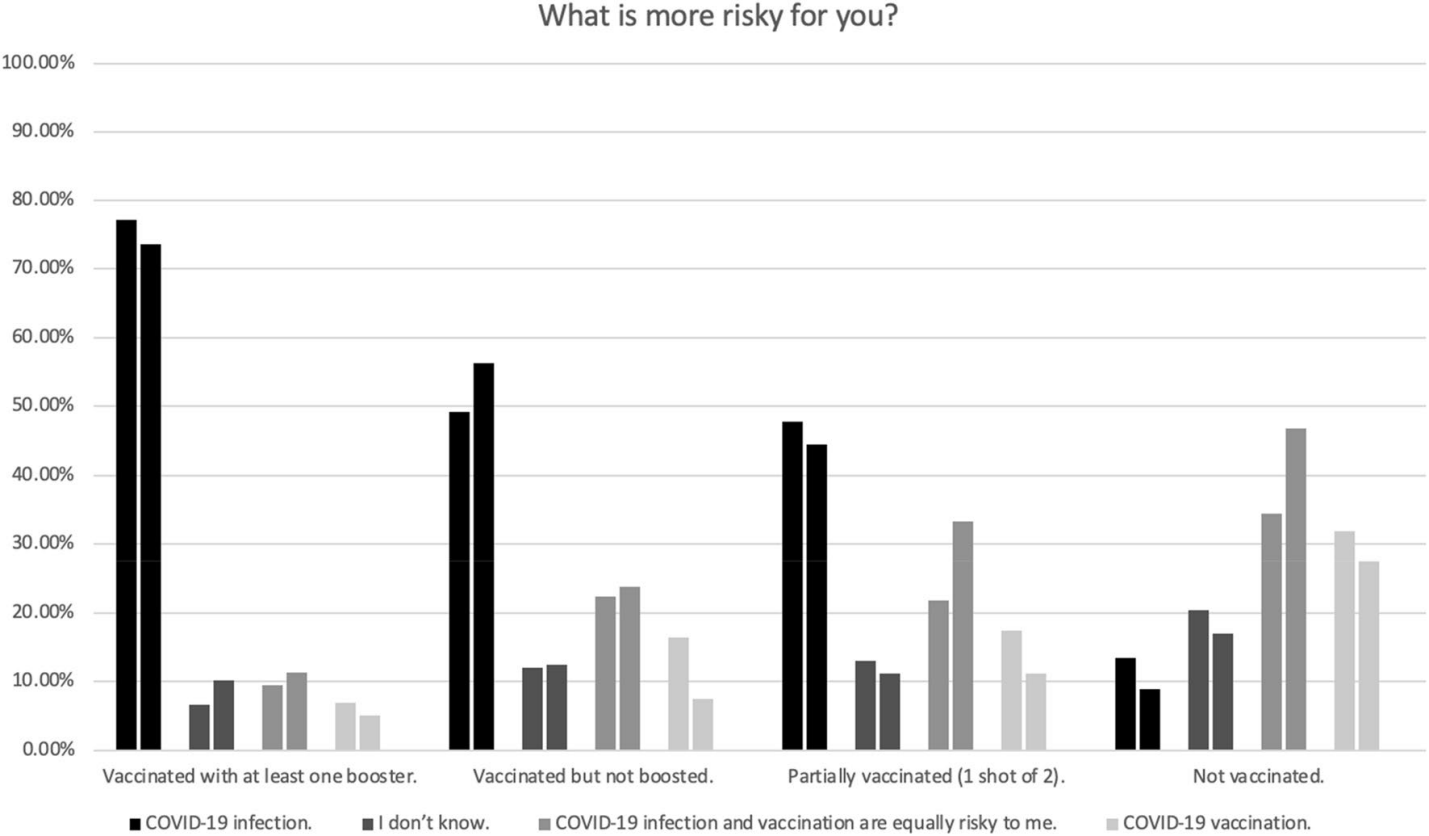
This figure shows the percentage of people with a given COVID-19 vaccination status who gave a particular answer to the question “What is more risky for you?” The left bar in each pair is from May 2022 (N = 400), and the right bar is from November 2022 (N = 615). There was a statistically significant relationship between risk perception and vaccination status, May, ${\chi }^{2}$ (9, N = 400) = 149.05, p < 0.001, W = 0.61; November, ${\chi }^{2}$ (9, N = 615) = 189.75, p < 0.001, W = 0.56.

Many participants dramatically overestimated the number of U.S. citizens who have died as a result of vaccination (Table 1). Figure 3 shows non-vaccinated participants are much more likely than vaccinated participants to think vaccination is equally or more risky than infection.

**Figure 4:**
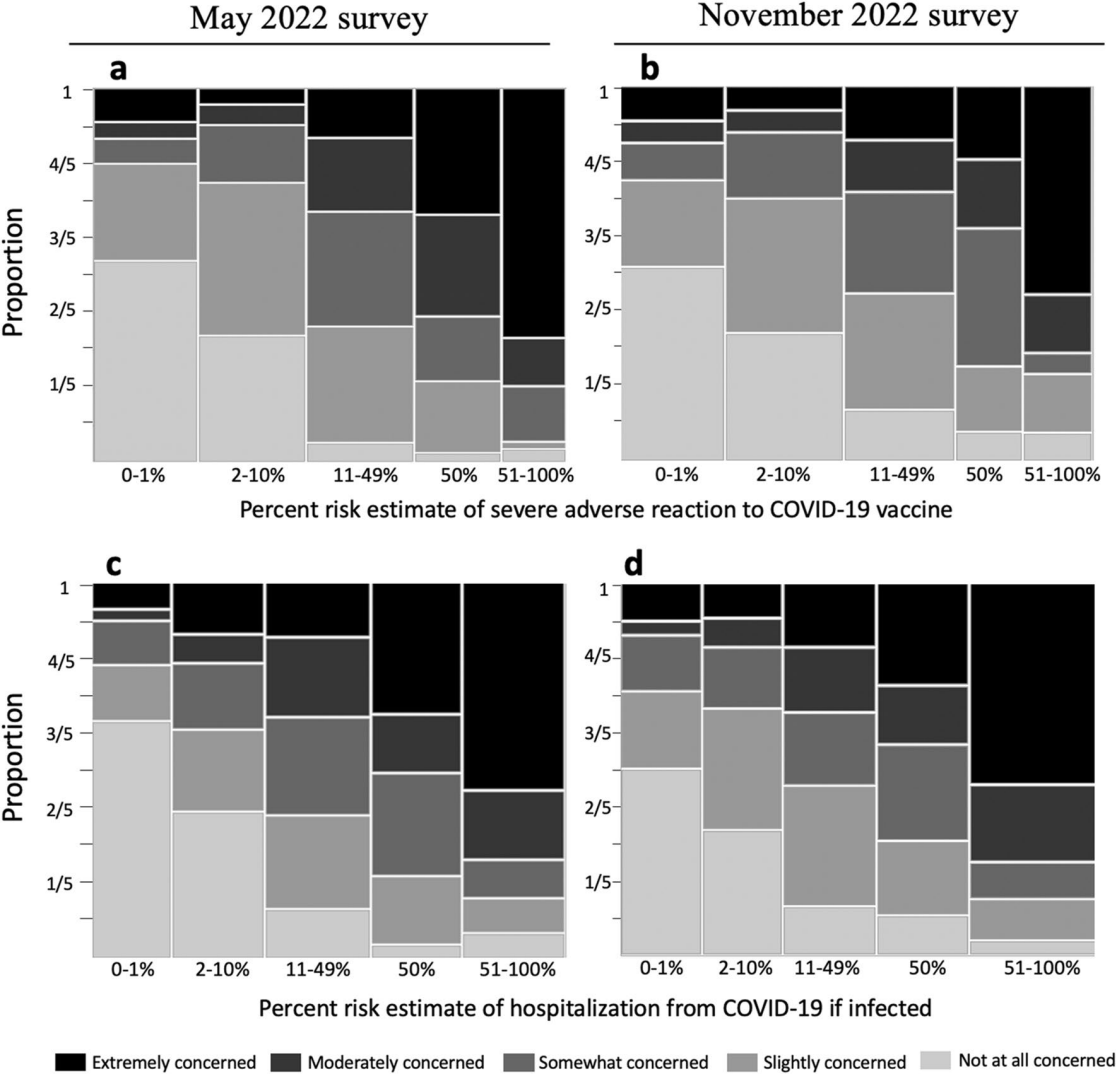
The figure shows the relationship between people’s percent risk estimates and their associated level of concern. We asked “If you receive a COVID-19 vaccine/booster in the future, what do you think your percent risk of a serious adverse reaction would be? An example of a serious adverse reaction is an allergic reaction requiring treatment in a hospital. Do not include your risk of common side effects such as fatigue” and “If you were infected with COVID-19 (without any vaccination), what do you think your percent risk of hospitalization from COVID-19 would be?” The width of the bars is proportional to the number of respondents who selected a response in each category. Figure d, for example, shows that more people selected a response between 51 and 100% than between 0 and 1%. The colors of the bar indicate the level of concern assigned by the participants for a given risk estimate. For example, in Figure d, half of people who estimated the risk of hospitalization from infection between 0–1% were “not at all concerned” (this is reflected in the light grey bar being a height of 1/2).

Most participants gave numerical risk estimates (due to both vaccination and infection) much higher than those estimated in peer-reviewed literature^37^ (Fig. 4). We also asked: “If you got a COVID-19 vaccine, how concerned would you be about a serious adverse reaction to the vaccine?” and “If you were infected with COVID-19, how concerned would you be about getting hospitalized?” The level of concern participants attached to a given risk estimate varied widely (Fig. 4). Many of the participants who estimated their risk of hospitalization if infected at 50% answered that they would be “not at all”, “slightly”, or “somewhat” concerned if infected. Risk estimates of 50% are common (50% Vaccination risk: May, *n* = 74/400; November, *n* = 115/615; 50% Infection risk: May, *n* = 77/400; November, *n* = 115/615). Some of these responses might be explained by prior research that found that people often give 50% probability estimates to indicate general uncertainty, whether that be uncertainty about specific frequencies or the belief that the event in question’s occurrence is up to chance^34,51^. In other words, someone estimating 50% does not necessarily mean that they believe that an event will occur roughly half the time^34,51^.

Although the median estimates for risk due to infection (May 26%; November 38%) were higher than median estimates for risk due to vaccination (May 20%; November 15%), the median responses were the same order of magnitude for both risks. In contrast, for an unvaccinated 40 year old person, the chance of hospitalization from COVID-19 infection (1.42%) is roughly 6000 times as large as the chance of a severe allergic reaction to the Pfizer COVID-19 vaccine (0.00021%)^37,77^.

### Risk perceptions are correlated with vaccination intent

**Figure 5:**
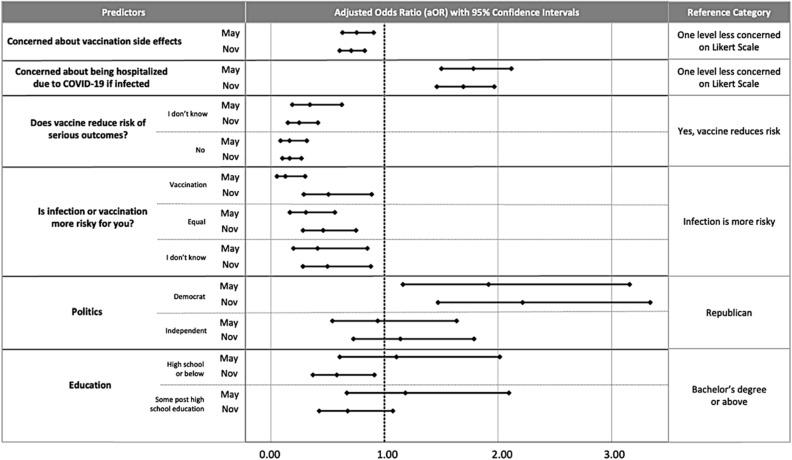
Correlates of COVID-19 vaccination intent include beliefs about the risks associated with COVID-19 vaccination and COVID-19 infection, beliefs about vaccine efficacy, political beliefs, and education. We chose the predictors in the initial full model and the reduced model presented based on the procedure described in the “Methods”. The multivariable ordinal logistic regression predicts vaccination intent, May, ${\chi }^{2}$ (11, N = 400) = 299.95, p < 0.001; November, ${\chi }^{2}$ (11, N = 615) = 386.83, p < 0.001. See Supp. Table 6 for exact values of aOR and 95% confidence intervals.

We quantified the impact of risk perception on vaccination intent while controlling for other variables commonly associated with vaccination intent (see Fig. 2 for intent question and control variables). The adjusted odds ratios shown in Fig. 5 tell us how many times as likely it is for someone with a given trait to be one level higher in vaccination intent than a person in the associated reference category.

Overall, participants’ beliefs about the risks and benefits of COVID-19 vaccination are correlated with future vaccination intent. Participants who said that vaccination is equally or more risky than infection were less sure about receiving a vaccine in the future than participants who said infection is riskier than vaccination. Consistent with these findings, participants who were concerned about severe adverse reactions to vaccination were less likely to intend to get more vaccination shots, and participants who were more concerned about COVID-19 infection were more likely to intend to be vaccinated in the future. Participants who did not think (or were not sure) that the vaccine reduces risk due to COVID-19 infection were less likely to intend to be vaccinated. The odds ratios show that intent is more strongly associated with beliefs about relative risk than intent is associated with education (which was only statistically significant in November). Additionally, there was no statistically significant association between intent and age, gender, ethnicity, or race (which were therefore removed from the models). See Fig. 5 and Supp. Table 6 for adjusted odds ratios.

### RRT impacted beliefs about relative risk

**Figure 6:**
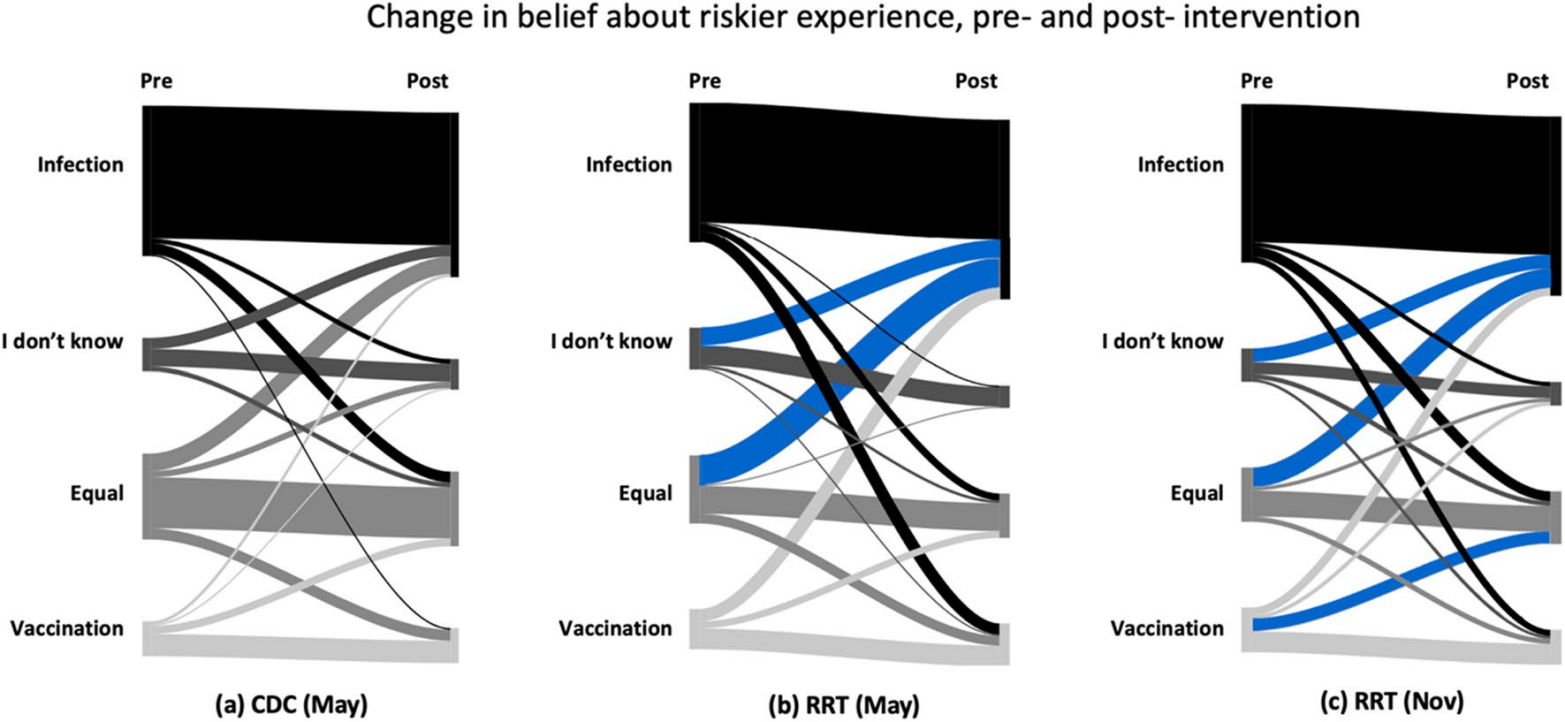
This diagram shows participants' responses to the question “What is riskier for you?” before and after seeing the CDC (**a**) and the RRT (**b**,**c**). Blue flows indicate a significant result at p < 0.05. Supplementary Tables 2 and 3 includes include statistics used to create diagram.

Respondents were asked to express their beliefs on whether COVID-19 vaccination or infection posed a higher risk for them. These beliefs were collected both before and after their visit to the CDC website or RRT tool (for questions, see Fig. 2, and for responses, see Fig. 6).

We used a McNemar–Bowker test to analyze symmetry in dependent responses^78^. A non-symmetrical response indicates a significant net change from one response pre-intervention to a different response post-intervention, i.e., that participants changed their mind in a specific direction after viewing the intervention. For respondents who viewed the CDC website in May, the global test was non-significant (*p* = 0.681) indicating symmetrical responses (i.e., the number of respondents who changed from one option to another was not significantly different than the converse). For respondents who viewed the RRT tool in May and November, the overall test was significant, and there was a medium effect size (May, *p* = 0.001, *g* = 0.23, 95% CI [0.17, 0.35]; November, *p* < 0.001, *g* = 0.16, 95% CI [0.11, 0.23]).

Post-hoc pairwise symmetry tests were conducted, and adjusted *p* values were calculated using the Benjamini–Hochberg procedure (see Fig. 6 and Supplementary Table 2). In Fig. 6, the blue flows indicate that a significantly greater number of participants changed their responses in the direction of the flow than the converse direction. In May for the RRT, the pairwise tests showed a significant change from participants selecting “COVID-19 infection and vaccination are equally risky” to “COVID-19 infection” as well as from “I don’t know” to “COVID-19 infection”. In November, there were significant changes from participants selecting “COVID-19 infection and vaccination are equally risky” to “COVID-19 infection,” from “I don’t know” to “COVID-19 infection”, and from “Vaccination is more risky” to “COVID-19 infection and vaccination are equally risky.” In May, 48.8% of participants thought infection was riskier than vaccination before seeing the RRT, and this changed to 62.0% post-intervention. In November, 54.5% thought infection was riskier than vaccination before seeing the RRT, and this changed to 61.6% post-intervention (see Supplementary Table 3 for confidence intervals for percentages).

### RRT increased intent to accept vaccination

**Figure 7:**
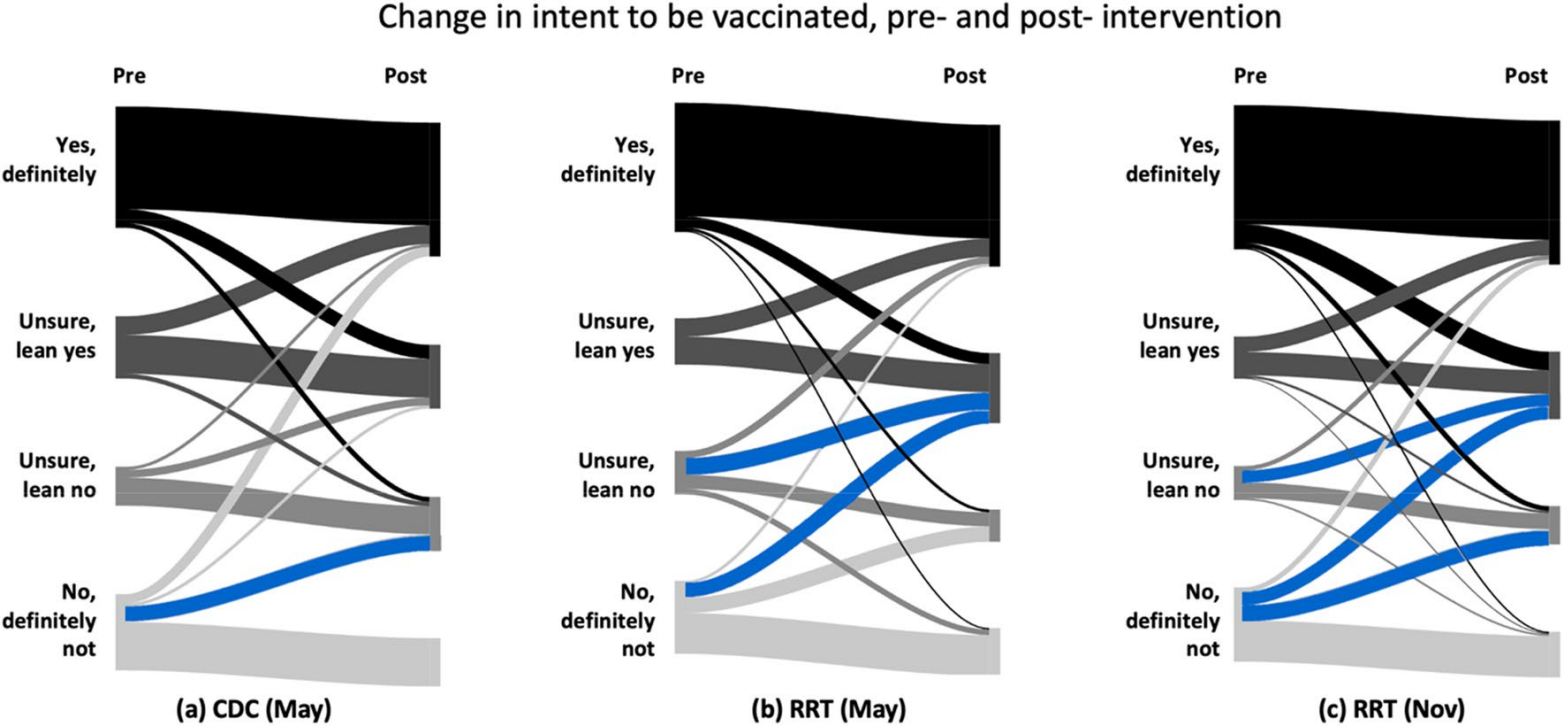
This figure shows citizens' responses to a question about intent to be vaccinated in the future before and after seeing the CDC (**a**) and the RRT (**b**,**c**) information about vaccination side effects and efficacy. Blue flows indicate a significant result at p < 0.05. Supplementary Tables 4 and 5 include statistics used to create diagram.

Before and after seeing information from either the CDC or RRT, we asked participants to rate their future intent to be vaccinated (Fig. 7). People who were already up to date on vaccinations were asked if they would get an additional booster if it was recommended. In a global test of symmetry, there were statistically significant effects of both the CDC intervention (*p* = 0.004, *g* = 0.23*,* 95% CI [0.17, 0.36]) and the RRT intervention (May, *p* < 0.001, *g* = 0.28, 95% CI [0.20, 0.39]); November, *p* < 0.001, *g* = 0.22, 95% CI [0.18, 0.29]).

Post-hoc pairwise symmetry tests were conducted and adjusted *p* values were calculated using the Benjamini–Hochberg procedure (see Fig. 7 and Supplementary Table 4). In Fig. 7 the blue flows are significantly larger than the corresponding flows in the converse direction. There was a significant change from participants selecting “No, definitely not,” before intervention to selecting “Unsure, lean no” after seeing the CDC information. After seeing the RRT, there was a significant change from selecting “No, definitely not,” to selecting “Unsure, lean yes” as well as from “Unsure, lean no” to “Unsure, lean yes” in both the May and November surveys. Additionally, in November there was a significant change from “No definitely not” to “Unsure, lean no” after seeing the RRT. After seeing the CDC information, the percentage of people who claimed they would “definitely not” be vaccinated decreased from 25.3 to 16.1%. After seeing the RRT, the percentage of people who claimed they would “definitely not” be vaccinated decreased from 25.3 to 16.3% in May and from 25.2 to 15.4% in December (see Supplementary Table 5 for confidence intervals for percentages). Although the RRT had a similar impact as the CDC on reduction in participants who responded “No, definitely not”, only participants exposed to the RRT had a statistically significant increase in responses of “Unsure, lean yes”.

## Discussion

It is important to empower people to assess the risks and benefits of vaccination in a way that is evidence-based, mathematically rigorous, and consistent with real-world data. Interventions must be designed with drivers of vaccine hesitancy in mind to be successful^15^. The RRT is tailored to address concerns about safety and efficacy, and we recommend using the RRT as part of an educational program that considers multiple factors driving vaccine hesitancy.

Consistent with previous studies^24^, the extent to which individuals perceived risks and benefits associated with vaccination emerged as an important factor in predicting COVID-19 vaccine hesitancy. The regression model predicted that people who were more concerned with risks associated with infection are more willing to accept future vaccinations, and people who are more concerned with the risks associated with vaccination are less willing to accept additional vaccinations. People who are uncertain or do not believe vaccination reduces the risk of serious outcomes from infection are less willing to receive future vaccinations. Further, many participants, including those with vaccinations, dramatically overestimated their percent risk of a serious adverse outcome to vaccination and the number of U.S. citizens who have died from vaccination. The combination of participants’ difficulties in assessing risk and the strength of the relationship between risk perceptions and vaccination intent suggests that education around risk has the potential to reduce vaccine hesitancy.

Because most people have limited ability to interpret percentages and decimals in health contexts, careful design of communication regarding numerical information is critical^66^. We designed the RRT to convey the relative risk of COVID-19 vaccination versus infection and the efficacy of vaccination in a way that allowed the general population to compare and comprehend the relative sizes of percentages. The CDC website also provides information about the efficacy of vaccination and the low risk posed by vaccination; however, the information often appears in formats (e.g., fractions and Cartesian coordinate systems) that are known in mathematics education to be hard to understand^30,32,79,80^. As such, it was not surprising that individuals shown the images from the CDC website did not have a statistically significant change in belief about relative risk even though their initial belief misaligned with the numerical information provided. In contrast, the RRT was more effective in changing participants’ beliefs about the relative risk of COVID-19 infection and vaccination. After seeing the RRT, there was a statistically significant shift from responding “infection and vaccination are equally risky” or “I don’t know” to “infection is riskier.”

Both the CDC and RRT had a positive effect on intent to accept COVID-19 vaccination. Even though both the CDC and the RRT conveyed that there are rare but serious risks associated with COVID-19 vaccination, there were no statistically significant decreases in intent to vaccinate. Both conveyed that vaccination reduced risk, but vaccinated individuals could still have severe outcomes from infection. This finding is useful because of the ethical importance of communicating honestly about the magnitude of a health intervention’s effectiveness along with the risks of an intervention to the public^81,82^.

It is challenging to design an online intervention that results in a change in vaccination intent, and many (but not all) interventions studied had no statistically significant impact or had impact only in some subgroups^75,83–88^. A few interventions had positive statistically significant impact on vaccine hesitancy^13,89–91^. A 2023 meta-analysis of 71 behavioral interventions targeting vaccine hesitancy found a small, but statistically significant, overall effect size^92^. Although the RRT and CDC information was effective at increasing intent to vaccinate, it did not significantly increase the number of people who were “definitely” intending to accept a vaccine. These gains in intention to vaccinate are still noteworthy for an online intervention that took participants on average between nine and ten minutes. The most effective educational interventions about vaccines used multiple strategies^15^, and we suspect, based on our interviews on the RRT, that one-on-one conversations using the RRT would better support decision making. Attempts to address misinformation about the COVID-19 vaccines have been most effective when using the following strategies: conveying the weight of evidence, utilizing humor, tailoring communications to target audiences, and incorporating warnings about misinformation^9^.

We also suspect that one-on-one conversations about relative risk with a trusted source could increase the impact of the RRT and CDC information because trust in government, health care providers, and the scientific process is related to vaccine hesitancy^93^. We acknowledge that mistrust in health care organizations and governments has a valid historical rationality, given prior U.S. government atrocities done in the name of medical research, especially against minoritized groups^93^. Although participants were provided links to the peer-reviewed sources on the RRT and told that the RRT appeared on national television and in the *Scientific American*, we understand that an online tool is not necessarily a highly credible source. After seeing either the CDC or RRT, we asked participants if the information seemed trustworthy. After seeing the RRT tool 81.7% (May) and 78% (November) responded “Yes, definitely” or “Unsure, lean yes.” In May, 72.4% of participants assigned to the CDC responded “Yes, definitely” or “Unsure, lean yes.” This study does not analyze the relationship between trust in information and change in intent after seeing information or the impact of information when conveyed by a trusted source; however, this is an important future consideration.

Several limitations impact the generalizability and applicability of research. The sample, while diverse, only includes U.S. adults with computer access who self-selected to participate in online surveys for monetary compensation. Although previously found to be reflective of the general U.S. population, research is ongoing about the representativeness of Prime Panels samples with regards to risk perceptions and health decision making^94^. Participants in our study provided similar percent risk estimates of hospitalization due to COVID-19 infection as participants in a larger, nationally representative survey of U.S. residents^95^. Paid online surveys allow for the collection of data from large groups, but it is impossible to guarantee all participants understood survey questions as intended or to draw strong conclusions about the reasons for their answers. Further, the COVID-19 global pandemic has been a continuously evolving health crisis since its onset in late 2019. The external influences on participants’ beliefs and decision making that we captured in our surveys are likely continually evolving, and this study does not attempt to quantify change in beliefs. We tested the RRT at two time points to gather stronger evidence of its effectiveness as opposed to studying change in beliefs over time. Finally, although the authors attempted to perform an unbiased a review of the data, our personal beliefs in the public good of widespread vaccination against COVID-19 may have introduced bias.

Early in the pandemic, a variety of risk calculators were rapidly created to help citizens understand COVID-19 risk and seen by millions of people^96–99^. Most widely-used COVID-19 risk calculators display people’s risk percentages without comparison to other risks. The risk percentages displayed are far lower than most U.S. residents’ estimates, and people struggle to make sense of small probabilities^44^. The research from this study of the RRT demonstrates how carefully communicating the relative risks of vaccination and infection, as well as designing communication tools with attention to prior research in mathematics education and risk communication, can reduce vaccine hesitancy.

Future directions for additional research are multifaceted. These include exploring the perceived trustworthiness of the RRT and the impact this has on effectiveness, testing the RRT in various real-world settings such as in-person with a healthcare provider, and measuring real-world outcomes to see if the RRT has an impact on the number of vaccinations received. It would be useful to compare the RRT across additional populations; a survey we conducted in South Korea showed the RRT had a larger impact in South Korea (in preparation). We also wonder about the impact of risk calculators portraying substantially lower risk estimates than users’ initial overestimates and whether this makes individuals more complacent about the risks of infection, as complacency about risk of disease is relevant to vaccine hesitancy^22^.

Because of the critical importance of vaccination for personal risk reduction and public health, alongside the ethical responsibility to disclose side effects of any medical intervention, additional research into and development of risk communication tools should be pursued.

## Methods

### Survey participants and sampling

Prime Panels are used to collect random human subjects data in multiple fields, including social and behavioral sciences^76,100^. By using Prime Panels, we were able to acquire a diverse and randomly selected sample from their participant pool, which is known to be reasonably representative of the U.S. population^76^. It should be noted that our sample had more women than the U.S. average, and a slightly different distribution of races than the U.S. as a whole. The average age of Prime Panel participants aligns with the U.S. average, and age significantly impacts COVID-19 risk. They were paid 1.50 USD for a survey that was completed on average in 9.1 min in May and 10.3 min in November. The payment, research procedures, and survey were approved by the University of Georgia Institutional Review Board with reference number PROJECT00002145. All research conducted followed the approved procedures and University of Georgia human subjects research guidelines and regulations. All participants were over 18 years old and gave informed consent before participating in research.

Surveying participants in May and November allowed us to determine if the RRT had a similar impact on a new sample of participants with 6 months more exposure to information. Public opinion about COVID-19 and its vaccine was highly politicized and shifted dramatically throughout the pandemic in the U.S.^101^. The safety and efficacy of a new bivalent booster was presented in Fall 2022^102^. Information about the risks of vaccination and new variants of COVID-19 were common in the news during those 6 months. For example, on October 7th, 2022 the state of Florida issued widely critiqued guidance recommending against COVID-19 mRNA vaccination in males ages 18 to 39 due to the risk of myocarditis caused by vaccination^103^.

### Survey questions

Participants took a 29-question online survey. Participants were asked if they had been infected with COVID-19, how many COVID-19 vaccinations (if any) they had, and why they were vaccinated (V-1 to V-2). Nine questions asked participants about their perceptions of risk associated with COVID-19 infection and vaccination including if they thought vaccination decreased risk due to infection (RP-0 to RP-8). We knew from pilot surveys that many participants thought vaccination and infection were equally risky, so we added follow up questions to see if they thought equal numbers of people have died due to vaccination and infection to better understand what they meant by “equally risky.” One question was a mathematical word problem about disease involving finding 10% of 1000 (RL-1), and six questions were about demographics (D-1 to D-6). As part of the survey, participants were randomly assigned to see information about risks and benefits of vaccination from either the CDC or the RRT and were asked questions related to the intervention (RRT-1 to RRT-11 or CDC-1 to CDC-12).

The survey questions were inspired by previous international surveys^8,75^, online pilot surveys with small samples^45,46^, and our interviews with U.S., South Korean, and Kenyan residents about COVID-19 infection and vaccination risk^36^. See Supplementary Table 7 for dates, sizes and citations associated with pilot surveys. The analysis of the pilot survey informed decisions on questions to include in the survey of a larger sample. We categorized open-ended responses to questions on the pilot surveys to create multiple-choice options on the survey for this study (Supplementary Figs. 1–3). We also used similar questions (RP-0, RP-2) to another research group^8,104^ who used an expert panel and comprehensive literature review to develop their 23-country survey on vaccine hesitancy. Their survey was used in the U.S. and informed by internally and externally validated surveys about COVID-19^105^. Additionally, some questions (V-1, RRT-11, and CDC-12) had similar wording as a prior study^75^ that asked participants in the U.S. and U.K. about intent to be vaccinated before and after seeing misinformation. To keep the survey to a manageable length, we intentionally focused our questions on participants’ perceptions of vaccine safety and efficacy and collected basic demographic information relevant to vaccine hesitancy (age, race, ethnicity, gender, education level, and political beliefs). Participants were allowed to select a race or races, ethnicity, and gender from multiple choice options or write in their preference. Multiple choice options were designed to be inclusive and use bias-free language as recommended by APA. Since prior research^8^ indicates that trust is an important factor in vaccine hesitancy, we asked a question about how trustworthy the participants found the CDC and the RRT information. Although access to vaccination is a critical global issue^106^, during the study period, COVID-19 vaccinations were provided free of charge by the U.S. government, regardless of insurance status, and thus we did not ask about access. We piloted the questions multiple times in interviews and small samples on Prime Panels and had experts in item design review the wording for clarity before the larger May and November samples. In order to use the most familiar wording, we used the term COVID-19 instead of SARS-COV-2 on survey questions.

### CDC and RRT intervention in survey

In May individuals were randomly assigned to see similar (but not identical) information presented by the CDC or RRT. Information from both the CDC and the RRT images stated that there are serious but extremely rare adverse events after vaccination, but each presented the information using different representations of data. We used the CDC estimates of the risk of particular adverse events in the design of the RRT and graphed the percentages provided by the CDC for comparison with other risks. We focused participants on particular parts of the CDC website or the RRT by embedding screenshots of the most relevant information into the survey and asking questions about the images before providing participants with the links to the content online. See images in survey instrument in supplementary materials. In addition to images, participants were provided with a short video about the RRT and a link to a video by the CDC.

Information from the CDC included graphs of the rates of COVID-19 hospitalizations for vaccinated and unvaccinated adults, statements about how many times as many unvaccinated adults were hospitalized due to COVID-19 as vaccinated adults, and information about rare side effects of vaccination such as anaphylaxis, thrombosis, myocarditis, and pericarditis. The CDC information said the vaccines were safe and effective and had been given to hundreds of millions of people, and the CDC also explained that the booster shots can enhance or restore protection that wanes after time. In addition to being provided with images of and links to the CDC website, participants were asked to answer questions about the CDC information to encourage reading the information and to check for comprehension. For example, we asked “According to the CDC, which is more risky for a young, healthy adult?” underneath a screenshot of relevant information. The graphics used by the CDC were significantly more complicated to understand than those recommended for use in health communication^39^.

Survey participants assigned to the RRT received a link to the full tool, but the survey also included specific screenshots of the RRT designed to highlight the relative risk of vaccination and infection and the reduction in risk due to vaccination. For example, we asked participants to compare the risk of death from COVID-19 if infected for a vaccinated and unvaccinated 76-year-old. The graphic included the absolute risk if infected but was designed to help people focus on relative risk. Another question asked participants to use an image of the RRT to compare the risk of hospitalization if infected with COVID-19 to the chance of a severe allergic reaction to the vaccine. Finally, we asked participants a question based on an image showing the risk of being hospitalized if infected for someone vaccinated over 6 months ago, someone vaccinated and boosted, and someone who is unvaccinated. Unlike the CDC website, the RRT provided no direct medical advice. The RRT did not say vaccination was safe and effective, provided no recommendations to get vaccinated or boosted, and did not recommend any particular behaviors with regard to mitigating COVID-19 infection risk.

After viewing the assigned intervention, we asked participants what they thought was riskier, if the information they saw was trustworthy, and whether they wanted to be vaccinated in the future. Based on these results, we found the RRT to be more effective than the CDC materials, so in November 2022, we assigned everyone to the RRT to gather further data about effectiveness.

### Refinement of the relative risk tool

In April and May of 2020, we interviewed 25 U.S. and 7 South Korean citizens about their mathematical understandings of quantitative representations of COVID-19 data that were ubiquitous in the media^36^. We conducted additional interviews from November 2020 to June 2022 and used a design-based research methodology^107^ to iteratively improve the RRT by incorporating feedback from the interviews and experts in public health and mathematics education. We conducted 14 interviews with U.S. residents, 10 with South Korean residents, and 9 with Kenyan residents. The interview sample was not representative, but it was diverse with respect to age, education, political affiliation, citizenship, income, and profession, which allowed us to iteratively improve the RRT. In the interviews we conducted after adding information about risks and efficacy of vaccination to the RRT, we found viewing the relative risk of vaccination versus infection and the reduction in risk due to vaccination resulted in people being more willing to be vaccinated.

We knew from prior research, interviews, and pilot surveys that citizens thought a risk of death of 1% or 2% was small, so we intentionally designed the RRT to help them understand the magnitude of those risks by comparing the risks to other situations. We knew from prior research^58^ and our interviews on RRT that some people think a decimal such as 0.0002 is larger than a decimal such as 0.02 because it has more digits, mirroring the fact that among whole numbers, larger numbers have more digits. Even highly educated people can struggle to determine if a 1, 5, or 10% risk of getting a disease is larger^59^. Previous research shows people especially struggle to make sense of low-probability risks^44^.

Estimating and communicating risks about COVID-19 accurately is complicated by several issues. One, the risk due to COVID-19 infection increases exponentially with age; for instance, an average 85-year-old is about 19,000 times as likely to die if infected than a 10-year-old^37^. Two, although it is possible to roughly compare the likelihood of *one* adverse outcome due to infection to *one* (different) adverse outcome due to vaccination, it is essentially impossible to compare the likelihood and seriousness of *all* risks posed by infection to *all* risks posed by vaccination. For instance, a severe allergic reaction to vaccination might be more or less serious than a hospitalization due to infection.

There are limitations of the statistical estimates provided on the RRT. Vaccination risk estimates are based on the information available and vary for different strains of SARS-COV-2, the differences of efficacy of vaccinations based on the strain, time since vaccination, and immune system response. COVID-19 infection risks are provided based on peer reviewed studies; however, the risk of infection changed over the course of the pandemic as knowledge about COVID-19 evolved and more treatment recommendations became available as well as with variations in local health care system capacity and preparedness. The risk percentages are averages, but there are differences in risk for people with various medical conditions, and only a few specific medical conditions are on the RRT. Further, it is much more challenging to quantify the likelihood and level of impact of non-acute risks such as long-COVID, the effectiveness of vaccination against long-COVID, and long-COVID-like symptoms triggered by vaccination^108–110^. The risk of death from COVID-19 is based on a particular population used to create the model and varies by access to medical care and general health of a population. In designing the RRT, we were constantly faced with the difficulty of providing risk estimates in an easy-to-understand manner, knowing that various peer-reviewed studies had different estimates.

### Statistics

We determined the sample size for the May 2022 survey using a power analysis for binary logistic regression. This was calculated with G*Power software. We chose a larger sample size in November because there were some interesting relationships in subgroups of original vaccination intent that we wanted to investigate in a larger sample. The sample size was much larger than needed to detect the relationships we focused on, but is not large enough to study the differential impact of RRT in different subgroups.

To create a model to predict vaccination status, which involves a series of vaccine shots, we employed use of ordinal logistic regression for each of the surveys. We used the model:

$logit\left(P\left(Y\le j\right)\right)={\beta }_{j0}+{\beta }_{j1}{x}_{1}\ldots {\beta }_{jp}{x}_{p}$ for $j=1,\dots ,J-1$ levels of the ordinal variable and *p* variables. Analyses were conducted using SPSS version 28. We first included responses to 13 survey questions as predictor variables in the model. Decisions about which question to include in the model were inspired from our pilot data, our observations in interviews, and from other research on vaccination hesitancy (see supplementary materials for the survey). The six survey questions about risk included questions about how concerned participants were about both vaccination and infection, what they thought was more risky, how many people they thought had died from either vaccination and from infection, and whether or not they thought the vaccination reduced risk of serious outcomes from infection. The six demographic predictors were race, ethnicity, age, gender, political party, and education level. Finally, we included responses to one question about previous COVID-19 infection as a predictor. To increase parsimony, we reduced the number of variables based on statistical significance in the first model and prior empirically demonstrated relationships (e.g., political beliefs and vaccination status). Relationships between intent to vaccinate and the independent variables are reported as adjusted odds ratios and 95% confidence intervals. The assumption of proportional odds was tested for each data set using the test of parallel lines. In each case, the test was not significant, indicating that the coefficients are the same across all response categories. The variance inflation factor (VIF) was calculated for each variable to test for multicollinearity. The assumption of no multicollinearity is met with VIF values below 3 for each variable.

The McNemar–Bowker test^78^ of symmetry was used to analyze the change in respondent’s perceptions of risk of vaccination and infection, as well as intent to receive a COVID-19 vaccination. These analyses were conducted using R 4.3.0 and the rcompanion package.

### Supplementary Information

Supplementary Information.

## Data Availability

The data from the May 2022 and November 2022 survey are available to download at http://www.covidtaser.com. Some participants took fewer than 3 min to complete the survey. After group discussion, the authors decide to exclude these responses from the analysis because such participants did not take sufficient time to read the questions and look at the CDC or RRT information.

## Code availability

The code for the McNemar–Bowker tests and the data used in SPSS 28 to create the regression model is available at http://www.covidtaser.com.
