# Supplementary material for: An effective COVID-19 vaccine hesitancy intervention focused on the relative risks of vaccination and infection
Source: Sci Rep. 2024 Mar 28;14:7419. doi: 10.1038/s41598-024-57841-1 (PMC10978892; doi:10.1038/s41598-024-57841-1)
Supplement: Supplementary file 1 — Supplementary Information. [file 41598_2024_57841_MOESM1_ESM.pdf]

Supplementary Information
for
An Effective COVID-19 Vaccine Hesitancy Intervention Focused on the Relative Risks of Vaccination and Infection

Cameron O'Neill Byerley, Dru Horne, Mina Gong, Stacy Musgrave, Laura A. Valaas, Brian Rickard, Hyunkyoung Yoon, Min Sook Park, Alison Mirin, Surani Joshua, Heather Lavender, Sukjin You

Supplementary Table 1. Sample characteristics of survey population by date.

	May 2022 CDC (%) N=192	May 2022 RRT (%) *N* = 208	Nov 2022 (%) *N* = 615
**Age Group**			
18-29	16.15	19.71	16.26
30-39	30.21	28.37	23.41
40-49	24.48	22.12	20.81
50-59	15.10	14.90	15.45
60+	13.02	12.02	20.81
No response	1.04	2.88	3.25
**Gender**			
Female	63.54	63.94	58.86
Male	33.85	36.06	38.54
Transgender, Non-Binary, or Prefer to not say	2.60	0.00	2.61
**Race**			
Black	20.83	15.87	17.40
Latino	0.52	1.44	2.60
White	71.88	75.96	70.89
Asian	2.08	2.88	2.76
Native Alaskan or American Indian	1.04	0.48	1.46
Native Hawaiian or Pacific Islander	0.52	0.48	0.16
More than one race	2.08	2.88	3.90
Prefer to not say	1.04	0.00	0.81
**Latino, Hispanic, or Spanish Origin**			
Yes	6.25	6.77	9.59
No	93.75	93.23	90.41
**Education**			
Less than 8th grade			0.16
Some High school, no diploma	3.13	6.73	6.83
High school graduate, diploma or equivalent	27.08	26.44	30.89
Some college credit, no degree	27.60	29.33	20.33
Trade/technical/vocational training	2.08	2.88	2.44
Associates degree	13.54	11.54	11.38
Bachelor's degree (for example: BA, BS)	19.79	17.79	18.05
Graduate degree	6.77	5.29	9.92
**Political stance**			
Democrat	31.25	28.85	34.31
Lean Democrat	14.06	11.54	9.11
Lean Republican	9.38	8.65	6.18
Republican	25.52	22.60	25.69
Independent	19.79	28.37	24.72
**Have you been infected with COVID-19?**			
Yes, and I tested positive	26.56	25.96	32.68
I had symptoms but no positive test	11.98	12.98	12.63
Not that I know of	61.46	61.06	54.96

Supplementary Table 2. Adjusted p values for McNemar-Bowker Test for RRT in May and November indicate differences in response pre and post intervention.

	May 2022 RRT				November 2022 RRT			
	Infection post	I don't know post	Equal post	Vaccine post	Infection post	I don't know post	Equal post	Vaccine post
Infection	--				--			
I don't know	.010*	--			.012*	--		
Equal	.010*	1	--		.043*	.814	--	
Vaccine	1	1	1	--	.814	.814	.043*	--

* $p < .05$, ** $p < .01$, *** $p < .001$, Adjusted p-values are calculated using the Benjamini-Hochberg procedure.

Supplementary Table 3. Change in U.S. residents' perception of relative risk after exposure to RRT information or CDC information.

	**In your personal opinion, what is more risky for you?**	**May 2022 (%) *N* = 400**	**Nov 2022 (%) *N* = 615**
**Pre-exposure**	COVID-19 infection	48.8 (43.9, 53.6)	54.5 (50.5, 58.4)
	I don't know	12.8 (10.0, 16.4)	11.4 (9.1, 14.1)
	Equal	25.7 (21.7, 30.3)	18.7 (15.8, 22.0)
	COVID-19 vaccination	12.8 (10,16.4)	15.4 (50.5, 58.4)
**Post-exposure to Relative Risk Tool (*n* = 208, *n* = 615)**	COVID-19 infection	62.0 (55.3, 68.3)	61.6 (57.7, 65.4)
	I don't know	7.7 (4.8, 12.1)	8.1 (6.2, 10.6)
	Equal	15.4 (11.1, 20.9)	18.2 (15.4, 21.5)
	COVID-19 vaccination	14.9 (10.7, 20.4)	12.0 (9.7, 14.8)
**Post-exposure to CDC information (*n* = 192)**	COVID-19 infection	53.6 (46.6, 60.6)	*N/A*
	I don't know	9.9 (6.4, 14.9)	*N/A*
	Equal	24.5 (18.9, 31.0)	*N/A*
	COVID-19 vaccination	12.0 (8.1, 17.3)	*N/A*

All numbers are percentages. In May 2022, each participant was randomly assigned to look at the Relative Risk Tool or selected CDC webpages on COVID-19 vaccination. Any respondent who spent less than 3 minutes taking the entire survey was removed from the data set, leading to slightly different numbers of people seeing the Relative Risk Tool (*n* = 208) as the CDC website (*n* = 192) in May.
Parentheses indicate 95% confidence intervals.

Supplementary Table 4. Adjusted p values for McNemar-Bowker Test for RRT in May and November indicate differences in vaccination intent pre and post intervention.

	May CDC (*N* = 192)				May RRT (*N* = 208)				November RRT (*N* = 615)			
	A	B	C	D	A	B	C	D	A	B	C	D
A. Yes, definitely	--				--				--			
B. Unsure, lean yes	.869	--			.540	--			.571	--		
C. Unsure, lean no	1	.869	--		.540	.009**	--		1	.001**	--	
D. No, definitely not	.124	.869	.027*	--	1	.013*	.242	--	.144	<.001***	<.001***	--

* $p < .05$, ** $p < .01$, *** $p < .001$, Adjusted p-values are calculated using the Benjamini-Hochberg procedure.

Supplementary Table 5. Exposure to RRT information increases intent to accept a COVID-19 vaccine relative to exposure to CDC information.

	Vaccination intent	May 2022 (%) *N* = 400	Nov 2022 (%) *N* = 615
**Pre-exposure**	Yes, definitely	42.5 (37.7, 47.4)	48.9 (45.0, 52.8)
	Unsure, lean yes	18.3 (14.8, 22.3)	14.3 (11.8,17.3)
	Unsure, lean no	14.0 (10.9, 17.7)	11.5 (9.3, 14.3)
	No, definitely not	25.3 (21.2, 29.7)	25.2 (21.9, 28.8)
**Post-exposure to Relative Risk Tool (*n* = 208, *n* = 615)**	Yes, definitely	48.6 (41.9, 55.3)	48.8 (44.8, 52.7)
	Unsure, lean yes	24.0 (18.7, 30. 3)	22.8 (19.6, 26.2)
	Unsure, lean no	11.1 (7.5, 16.0)	13.0 (10.6, 15.8)
	No, definitely not	16.3 (11.9, 22.0)	15.4 (12.8, 18.5)
**Post-exposure to CDC information (*n* = 192)**	Yes, definitely	44.7 (37.9, 51.8)	*N/A*
	Unsure, lean yes	21.3 (16.1, 27.7)	*N/A*
	Unsure, lean no	17.7 (13.0, 23.7)	*N/A*
	No, definitely not	16.1 (11.6, 22.0)	*N/A*

All numbers are percentages. In May 2022, each participant was randomly assigned to look at the Relative Risk Tool or selected CDC webpages on COVID-19 vaccination. Any respondent who spent less than 3 minutes taking the entire survey was removed from the data set, leading to slightly different numbers of people seeing the Relative Risk Tool (*n* = 208) as the CDC website (*n* = 192) in May.

Parentheses indicate 95% confidence intervals. The question about vaccination intent was an intentional replication of a question from Loomba et al. (2021) to allow comparison of vaccination intent in the U.S. in September, 2020 reported by Loomba to May and November, 2022.

Supplementary Table 6. Correlates of COVID-19 vaccination intent (Yes, definitely; Unsure, lean yes; Unsure, lean no; No, definitely not) with beliefs about the risks associated with COVID-19 vaccination and COVID-19 infection, beliefs about vaccine efficacy, political beliefs and education.

	Adjusted odds ratio		95% Confidence interval		SE		Wald Chi-Square		Sig.	
	May	Nov	May	Nov	May	Nov	May	Nov	May	Nov
**Concerned about vaccination side effects** (ref = one level less concerned on Likert Scale)	0.76	0.71	(0.63, 0.91)	(0.61, 0.83)	0.09	0.08	9.23	19.01	**	***
**Concerned about being hospitalized due to COVID-19 if infected** (ref = one level less concerned on Likert Scale)	1.78	1.70	(1.50, 2.12)	(1.46, 1.97)	0.09	0.08	43.61	48.55	***	***
**Does vaccine reduce risk of serious outcomes?** (ref = yes)										
I don't know	0.34	0.25	(0.19, 0.63)	(0.15, 0.42)	0.30	0.26	12.30	28.75	***	***
No	0.17	0.17	(0.09, 0.32)	(0.10, 0.27)	0.33	0.25	29.35	53.10	***	***
**Is infection or vaccination more risky for you?** (ref = infection)										
Vaccination	0.13	0.51	(0.06, 0.30)	(0.29, 0.89)	0.44	0.28	22.17	5.66	***	*
Equal	0.31	0.46	(0.17, 0.56)	(0.28, 0.75)	0.31	0.25	14.62	9.70	***	**
I don't know	0.41	0.50	(0.20, 0.85)	(0.28, 0.88)	0.37	0.29	5.76	5.75	*	*
**Political party** (ref = Republican)										
Democrat	1.92	2.22	(1.16, 3.16)	(1.47, 3.34)	0.26	0.21	6.55	14.53	*	***
Independent	0.94	1.14	(0.54, 1.64)	(0.73, 1.79)	0.28	0.23	0.05	0.33		
**Education Level** (ref = completed bachelor's degree or higher)										
Some post high school education	1.11	0.58	(0.61, 2.02)	(0.37, 0.91)	0.31	0.23	0.11	5.57		*
High school or below	1.19	0.68	(0.67, 2.10)	(0.43, 1.07)	0.29	0.24	0.34	2.74		

* $p < .05$, ** $p < .01$, *** $p < .001$.

Note: Adjusted odds ratios (aOR) and 95 percent CI were calculated using ordinal logistic regression. Reference categories: not concerned about COVID-19 vaccination, not concerned about COVID-19 infection, believes vaccine reduces risk of COVID-19 infection, believes COVID-19 infection is riskier than COVID-19 vaccination for them, Republican, completed bachelor's degree or higher.

Supplementary Table 7. Timeline of Development of RRT and Pilot Surveys

Date	Activity	Citations
**April & May, 2020**	Interviewed U.S. and South Korean about COVID-19 data. Interviews included questions about risk of severe outcomes. Qualitative analysis of interviews inspired creation of RRT.	Yoon, H. *et al.* United States and South Korean citizens' interpretation and assessment of COVID-19 quantitative data. *The Journal of Mathematical Behavior* **62**, 100865 (2021).
**August, 2020 to May 2022**	Designed and refined the RRT.<br><br>We updated it to improve areas of confusion then retested in new interviews in U.S., South Korea and Kenya, sent it to experts for review, presented on the RRT at conferences, taught lessons using the RRT in schools, analyzed New York Times reader's comments, and added new peer-reviewed data as a result of requests for particular comparisons.	Joshua, S., Drimalla, J., Horne, D., Lavender, H., Yon, A., Byerley, C., ... & Moore, K. (2022). Exploring Relative Size with Relative Risk. *Mathematics Teacher: Learning and Teaching PK-12*, *115*(5), 339-350.<br><br>See www.covidtaser.com for list of conference talks.<br><br>Link to New York Times comments on RRT: https://www.nytimes.com/2022/04/17/science/covid-risks.html
**Sept. 2021 to May 2022**	May 13, 2022: Pilot survey of 51 U.S. residents.<br>Feb. 8, 2022: Pilot survey of 63 U.S. residents.<br>Jan 29, 2022: Pilot survey of 104 U.S. residents.<br>Dec 13, 2021: Pilot survey of 49 U.S. residents.<br>Sep 16, 2021: Pilot survey of 49 U.S. residents.<br><br>The questions on pilot surveys were very similar to the final surveys reported in this paper.<br><br>We used pilot surveys to collect open-ended responses to find out why people picked particular options and made multiple-select options by categorizing those responses.<br><br>We made minor changes to some pilot survey questions to improve wording based on initial data, expert review, and conference feedback. We removed some survey items that did not give as interesting of data to make the overall survey length reasonable and to encourage people to read each question.<br><br>The peer-reviewed conference papers report pilot survey results.	Yoon, H. et al. in *44th Annual Meeting of the North American Chapter of the International Group for the Psychology of Mathematics Education*. (eds A. Lischka et al.) 862-871.<br><br>Gong, M., Horne, D., Yoon, H. & Byerley, C. in *25th Annual Conference on Research in Undergraduate Mathematics Education*.

Supplementary Figure 1. US citizens who believe that a COVID-19 infection is more risky than a COVID-19 vaccination selected the following reasons.

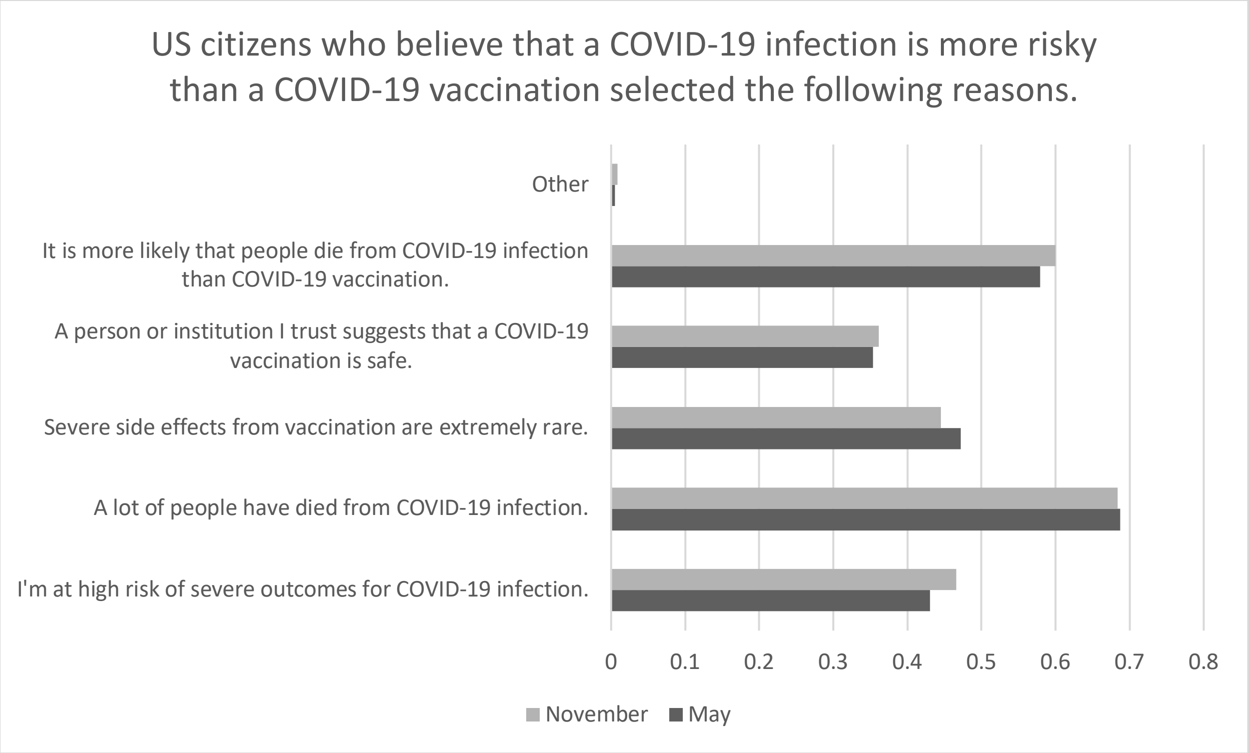

U.S. residents who thought infection was riskier than vaccination were asked to choose (or write in) their reasons. They were allowed to select multiple answers. The chart shows the proportion of people who thought infection is riskier than vaccination who gave each response.

Other Responses:
"I don't think the vaccine is effective."
"No risk."
"Compromised immune system."

Supplementary Figure 2. US citizens who believe that COVID-19 infection is equally risky as a COVID-19 vaccination.

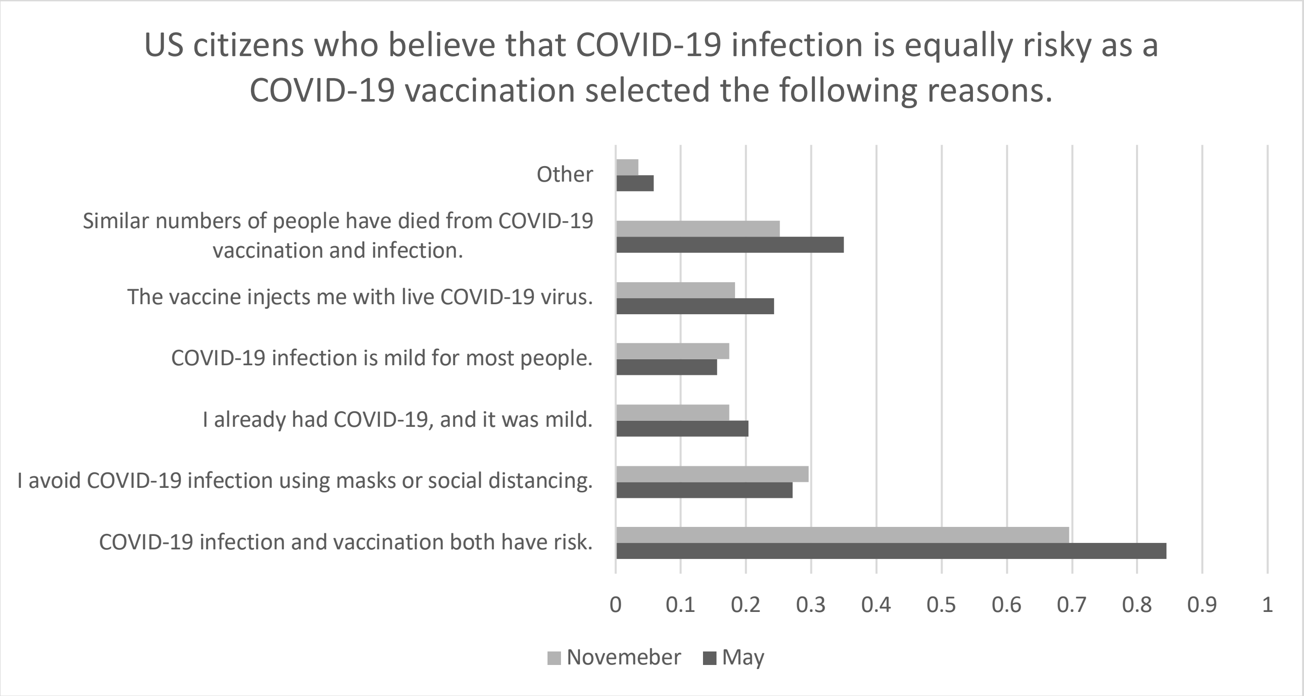

U.S. residents who thought infection is equally risky as vaccination were asked to choose (or write in) their reasons. They were allowed to select multiple answers.

Other Responses:
"Testing wasn't long enough for me to be comfortable with the results of studies."
"I have pre-existing medical conditions that make both equally dangerous for me."
"I am not at risk and I do not intend to get vaccinated."
"I know someone with long covid. It is not fun. Also, I was given the booster wrong and I am still suffering from that."
"The vaccine does not work."
"My mother had a major reaction to a booster. She had both kidneys shutdown, heart failure, brain hematoma."
"The government is hiding much of the truth and banning opinions contrary to what they want."
"Makes no sense. Why be trusting of a government who produced a MAN MADE VIRUS and then be asked to trust the same government with a MAN MADE VACCINE ???? NO Thanks."
"Personal opinion I don't want to screw with my natural immune system."
"I can't get the vaccine because I have GBS and my doctor won't chance the effects."

Supplementary Figure 3. US citizens who believe that COVID-19 infection is less risky than a COVID-19 vaccination selected the following reasons.

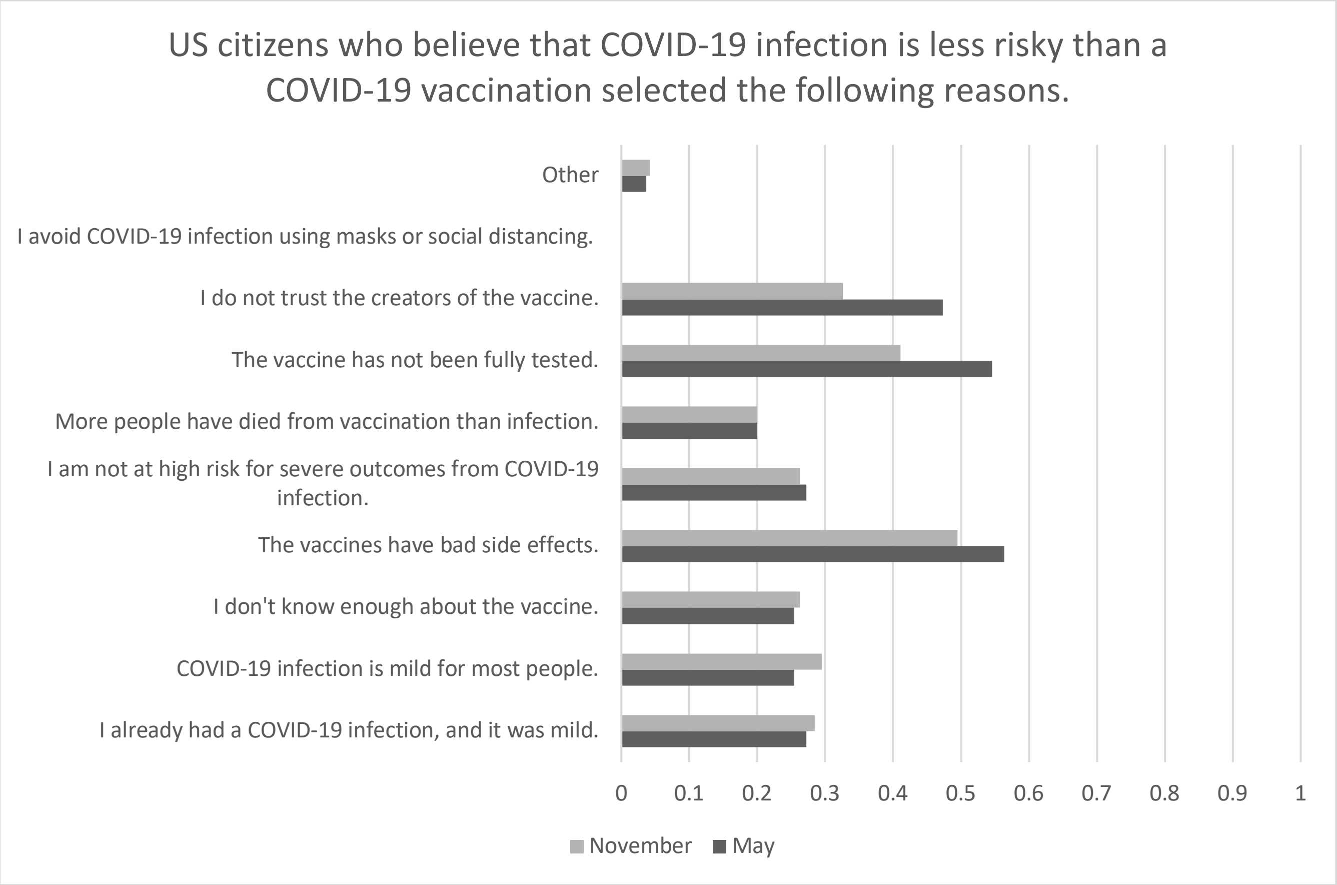

U.S. residents who thought infection is less risky than vaccination were asked to choose (or write in) their reasons. They were allowed to select multiple answers.

Other responses:
"These shots are not vaccines. The Moderna shot killed my father-in-law."
"My immune system is excellent."
"Everyone I know who has been vaccinated and has boosters have gotten covid more than once."
"I just won't do it."
"Two family members died after the vaccine. They were otherwise healthy with no underlying conditions."
"Against vaccines all together."

Survey Instrument

RAPID: Creating Opportunities for Visualization of Data: Applying STEM Education Research

1. Introduction

You are being asked to take part in a research study. The information in this form will help you decide if you want to be in the study. Please ask the PI (Dr. Byerley) and other researcher(s) below if there is anything that is not clear or if you need more information. The research is funded by the National Science Foundation RAPID program Award # DUE: 2032688.

We are doing this research study to learn more about how people understand data related to COVID-19. You are being invited to be in this research study because you are participating in the Prime Panels. If you agree to participate in this study, we will ask you to look at websites with mathematical information about COVID-19. We will ask you questions about what you understand from the data.

It will take about 10 minutes. Participation is voluntary. You can refuse to take part or stop at any time without penalty. Upon completion of the study, you will receive compensation in the amount you have agreed to with the platform through which you entered this survey. There are questions that may make you uncomfortable because they are related to risk of death from various activities.

Your responses may help us understand how adults understand the unfolding coronavirus situation. Your responses might help us make recommendations for communication on coronavirus with the general population.

This study is a collaboration between researchers at the University of Georgia, Arizona State University, Louisiana State University, and University of Wisconsin Milwaukee. Data from the research will be shared between the research teams. This research involves the transmission of data over the Internet. Researchers do not have any access to your Personally Identifiable Information on Prime Panels. Please feel free to ask questions about this research at any time.

You can contact the Principal Investigator, Dr. Byerley at cbyerley@uga.edu. If you have any complaints or questions about your rights as a research volunteer, contact the IRB at 706-542-3199 or by email at IRB@uga.edu.

Principal Investigator: Cameron Byerley University of Georgia cbyerley@uga.edu
Co-Investigator: Kevin Moore University of Georgia kvcmoore@uga.edu
Senior Personnel: Hyunkyoung Yoon Arizona State University hyoon14@asu.edu
Senior Personnel: Surani Joshua Arizona State University sjoshua@asu.edu
Consultant: Min Sook Park University of Wisconsin-Milwaukee minsook@uwm.edu
Consultant: Heather Lavender Louisiana State University heatherl@lsu.edu

If you agree to participate in this research study, please check the box saying you consent to the research.
Do you agree with the consent form?

- O Yes
- O No

2. Vaccine Status

V-1. Have you had a COVID-19 vaccine? [Depending on the participant's response they were given slightly different wording for the follow up questions.]

- O Yes, I am up to date on vaccination for COVID-19 (two doses of Pfizer or Moderna, or single dose of J&J vaccine) including at least one booster shot.
  - *V-1-a1. You said you have been boosted. If recommended, do you intend to ge t another COVID-19 booster shot in the future?*
    - O Yes, definitely.
    - O Unsure, lean yes.
    - O Unsure, lean no.
    - O No, definitely not.
  - *V-1-a2. You said you have been vaccinated and boosted. What was your primary reason for being vaccinated?*

- I wanted to be vaccinated.
    - My employer required me to.
    - Someone else pressured me to do it.
    - Vaccination was required for something I wanted to do.
    - Other: ____
- Yes, I am vaccinated for COVID-19 (two doses of Pfizer or Moderna, or single dose of J&J vaccine) but did NOT receive a booster shot.

  V-1-b1. You said you have not been boosted. Do you intend to get a COVID-19 booster shot in the future?
    - Yes, definitely.
    - Unsure, lean yes.
    - Unsure, lean no.
    - No, definitely not.

  V-1-b2. You said you have been vaccinated. What was your primary reason for being vaccinated?
    - I wanted to be vaccinated.
    - My employer required me to.
    - Someone else pressured me to do it.
    - Vaccination was required for something I wanted to do.
    - Other: ____
- Yes, I am partially vaccinated for COVID-19 (one dose of Pfizer or Moderna).

  V-1-c1.You said you currently have been partially vaccinated. Do you intend to get another dose of a COVID-19 vaccine in the future?
    - Yes, definitely.
    - Unsure, lean yes.
    - Unsure, lean no.
    - No, definitely not.

  V-1-c2.You said you currently have been partially vaccinated. What was your primary reason for being vaccinated?
    - I wanted to be vaccinated.
    - My employer required me to.
    - Someone else pressured me to do it.
    - Vaccination was required for something I wanted to do.
    - Other: ____
- No [respondent was not vaccinated].

  V-1-d.You said you currently have not been vaccinated. Do you intend to get a COVID-19 vaccine in the future?
    - Yes, definitely.
    - Unsure, lean yes.
    - Unsure, lean no.
    - No, definitely not.

Vaccination intent questions are updated versions of items and item choices from (Loomba et al., 2021).

V-2. Have you been infected with COVID-19 at any time?
- Yes, and I tested positive.
- I had symptoms of COVID-19 but did not have a positive test, so I'm not sure.
- Not that I know of.

3. Risk Perception

RP-0. The risks of COVID-19 disease are greater than the risks of the vaccine. Item from (Lazarus et al., 2022).
- Strongly agree
- Somewhat agree
- Unsure/no opinion
- Somewhat disagree
- Strongly disagree

RP-1. In your ***personal opinion****, what is* ***more risky*** *for* ***you****? [Depending on the participant's response they were given slightly different wording for the follow up question.]*

- COVID-19 infection.
  - *RP-1-a. On a prior survey, US citizens who believe that a COVID-19 infection is* ***more risky*** *than a COVID-19 vaccination gave the following reasons. Select all of the reasons you agree with.*
    - I'm at high risk of severe outcomes for COVID-19 infection.
    - A lot of people have died from COVID-19 infection.
    - Severe side effects from vaccination are extremely rare.
    - A person or institution I trust suggests that a COVID-19 vaccination is safe.
    - It is more likely that people die from COVID-19 infection than COVID-19 vaccination.
    - Other: ______________________________
- COVID-19 infection and vaccination are equally risky to me.
  - *RP-1-b. On a prior survey, US citizens who believe that COVID-19 infection is* ***equally risky*** *as a COVID-19 vaccination gave the following reasons. Select all of the reasons you agree with.*
    - COVID-19 infection and vaccination both have risk.
    - I avoid COVID-19 infection using masks or social distancing.
    - I already had COVID-19, and it was mild.
    - COVID-19 infection is mild for most people.
    - The vaccine injects me with live COVID-19 virus.
    - Similar numbers of people have died from COVID-19 vaccination and infection.
    - Other: ______________________________
- COVID-19 vaccination.
  - *RP-1-c. On a prior survey, US citizens who believe that COVID-19 infection is* ***less risky*** *than a COVID-19 vaccination gave the following reasons. Select all of the reasons you agree with.*
    - I already had a COVID-19 infection, and it was mild.
    - COVID-19 infection is mild for most people.
    - I don't know enough about the vaccine.
    - The vaccines have bad side effects.
    - I am not at high risk for severe outcomes from COVID-19 infection.
    - More people have died from vaccination than infection.
    - The vaccine has not been fully tested.
    - I do not trust the creators of the vaccine.
    - I avoid COVID-19 infection using masks or social distancing.
    - Other: ______________________________
- I don't know.

RP-2. In your ***personal opinion****, does the COVID-19 vaccine* ***reduce*** *the risk of serious COVID-19 infection outcomes for* ***you****?*

- Yes.
- No.
- I don't know.

RP-3. If you were infected with COVID-19 (without any vaccination), what do you think your percent risk of hospitalization from COVID-19 would be?
(Enter number between 0 and 100 without % sign. For example, 0.05, 2, 27, 50.5)

RP-4. If you were infected with COVID-19, how concerned would you be about getting hospitalized?

- Not at all concerned.
- Slightly concerned.
- Somewhat concerned.
- Moderately concerned.
- Extremely concerned.

RP-5. If you receive a COVID-19 vaccine/booster in the future, what do you think your percent risk of a serious adverse reaction would be?
An example of a serious adverse reaction is an allergic reaction requiring treatment in a hospital. Do not include your risk of common side effects such as fatigue. (Enter number between 0 and 100 without % sign.)

RP-6. If you got a COVID-19 vaccine, how concerned would you be about a serious adverse reaction to the vaccine?

- ○ Not at all concerned.
- ○ Slightly concerned.
- ○ Somewhat concerned.
- ○ Moderately concerned.
- ○ Extremely concerned.

RP-7. To your knowledge, approximately ***how many*** *US citizens have died primarily because of COVID-19* ***vaccination****? (As of November 2022).*

- ○ I do not think anyone has died due to COVID-19 vaccination.
- ○ Less than 50 people.
- ○ Between 50 and 500 people.
- ○ Between 500 and have 50,000 people.
- ○ Between 50,000 and 500,000 people.
- ○ Over 500,000 people have died due to vaccination.

RP-8. To your knowledge, approximately ***how many*** *US citizens have died primarily because of COVID-19* ***infection****? (As of November 2022).*

- ○ I do not think anyone has died due to COVID-19 infection.
- ○ Less than 50 people.
- ○ Between 50 and 500 people.
- ○ Between 500 and 50,000 people.
- ○ Between 50,000 and 500,000 people.
- ○ Over 500,000 people have died due to infection.

4. Risk Literacy

RL-1. If the chance of getting a disease is 10%, how many people would be expected to get the disease ***out of 1000****?*

5. Relative Risk Tool or CDC Intervention (In May participants were randomly assigned to explore either Relative Risk Tool or CDC information. In November they were all assigned to the RRT.)

5-1. Relative Risk Tool

RRT-1. Please watch this video about the Relative Risk Tool: https://www.youtube.com/watch?v=9Yn3wIDLq1M
The Relative Risk Tool was created by a medical doctor and mathematicians to help citizens make sense of risk and make personal choices about COVID-19 infection and vaccination. It was featured in Scientific American and on TV, and translated to Korean.

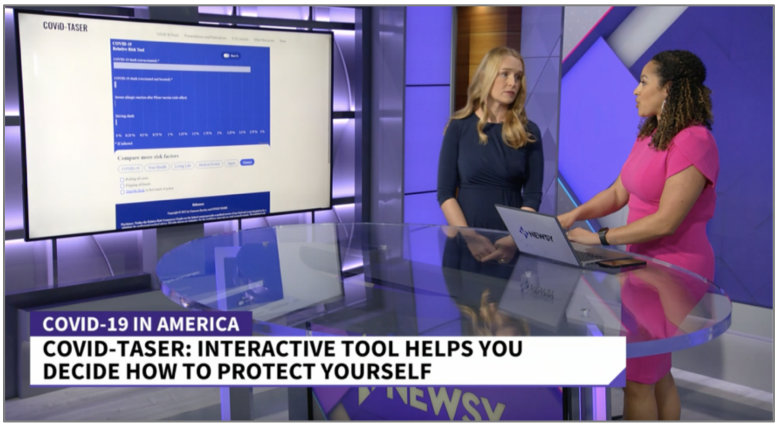

Please open [the Relative Risk Tool](), explore it, and come back.

- ○ I have watched the video and explored the Relative Risk Tool.

*RRT-2. **According to the Relative Risk Tool**, which is more risky?*

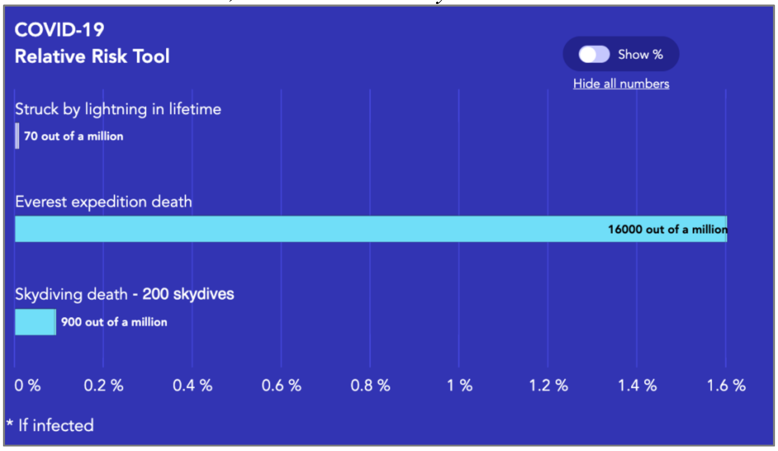

- O 200 skydives.
- O Everest expedition.
- O They are equally risky.
- O I don't know.

*RRT-3. Fill in the blank according to your **personal opinion**:*
An unvaccinated 76-year-old is ____________ to die from a COVID-19 infection than a vaccinated 76-year-old.

- O more likely
- O equally likely
- O less likely

*RRT-4. **According to the Relative Risk Tool image below**, which is most risky for a 76-year-old?*

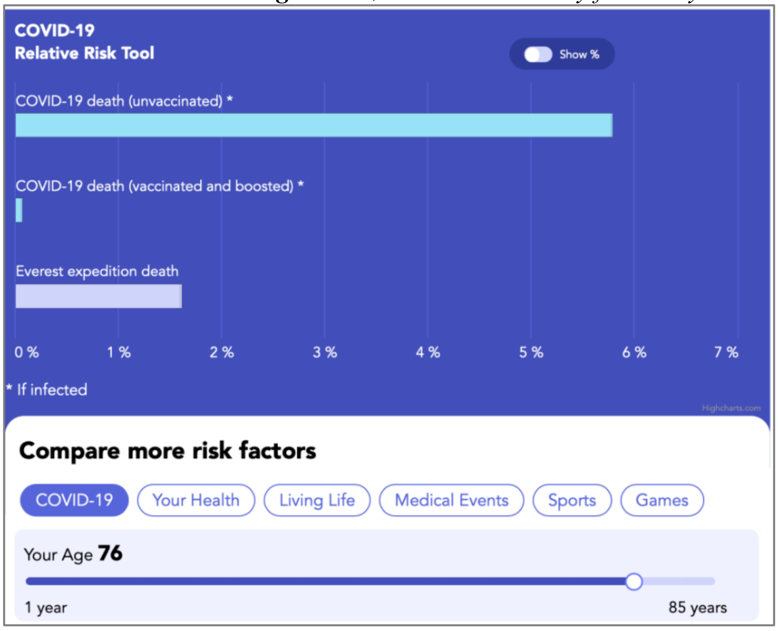

- O COVID-19 infection while vaccinated and boosted.
- O COVID-19 infection while unvaccinated.
- O An Everest expedition.
- O I don't know.

*RRT-5. In your **personal opinion**, which is more risky for an average 27 year old?*

- O A COVID-19 infection.
- O A COVID-19 vaccination.
- O COVID-19 vaccination and infection are equally risky.
- O I don't know.

RRT-6. ***According to the Relative Risk Tool****, which is more risky for an average 27 year old?*

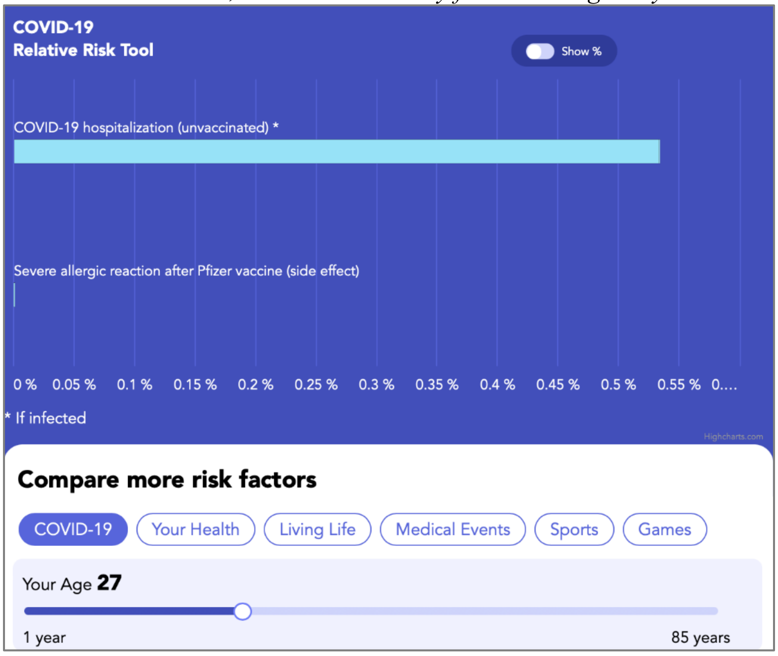

- ○ COVID-19 infection.
- ○ COVID-19 vaccination.
- ○ COVID-19 infection and vaccination are equally risky.
- ○ I don't know.

RRT-7. ***According to the Relative Risk Tool****, does the COVID-19 booster vaccination* ***reduce*** *the risk of hospitalization from COVID-19 infection?*

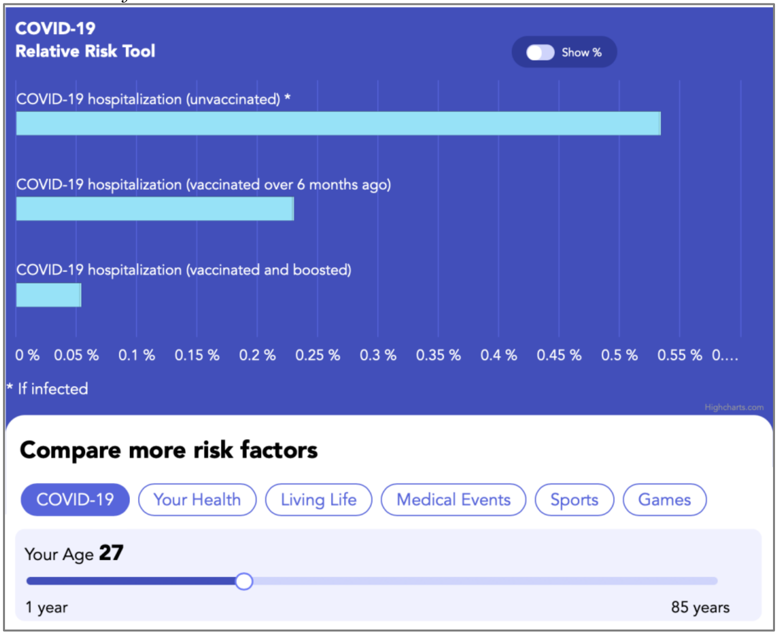

- ○ Yes.
- ○ No.
- ○ I don't know.

RRT-8. Click the references at the bottom of the Relative Risk Tool to see where the data comes from. After reviewing the references, does the Relative Risk Tool seem ***trustworthy****?*

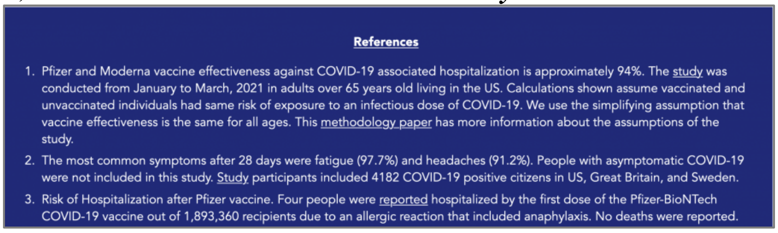

- ○ Yes, definitely.
- ○ Unsure, lean yes.
- ○ Unsure, lean no.
- ○ No, definitely not.

RRT-9. After using the Relative Risk Tool, what do you believe is ***more risky*** *for you?*

- ○ COVID-19 infection.
- ○ COVID-19 vaccination.
- ○ COVID-19 infection and vaccination are equally risky.
- ○ I don't know.

RRT-10. After using the Relative Risk Tool, do you think a COVID-19 vaccine is more or less risky than you did before?

- ○ More risky.
- ○ Equally risky.
- ○ Less risky.

RRT-11. After using the Relative Risk Tool, do you intend to get a COVID-19 vaccine in the future? Or, if already vaccinated, do you intend to get a first or second booster if it is recommended?

- ○ Yes, definitely.
- ○ Unsure, lean yes.
- ○ Unsure, lean no.
- ○ No, definitely not.

5-2. CDC (Half of participants were randomly assigned to CDC questions in May, 2022)

CDC-1. Visit the CDC's webpage Hospitalization by Vaccination Status and view the graphs.

- ○ I have visited and viewed graphs on the CDC website.

CDC-2. Fill in the blank according to your ***personal opinion****:*
An unvaccinated 76-year-old is ____________ to be hospitalized from a COVID-19 infection than a vaccinated 76-year-old.

- ○ More likely
- ○ Equally likely
- ○ Less likely

*CDC-3. **According to the CDC image below**, who is most likely to be hospitalized from COVID-19 infection?*

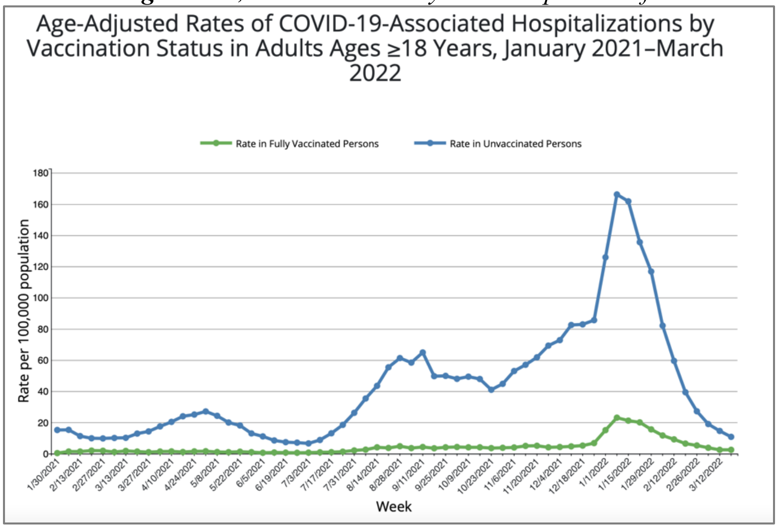

- ○ Fully vaccinated adult.
- ○ Unvaccinated adult.
- ○ I do not know.

*CDC-4. Fill in the blank **according to the CDC information below**.*
An unvaccinated person is ____________ to be hospitalized from COVID-19 infection than a vaccinated person.

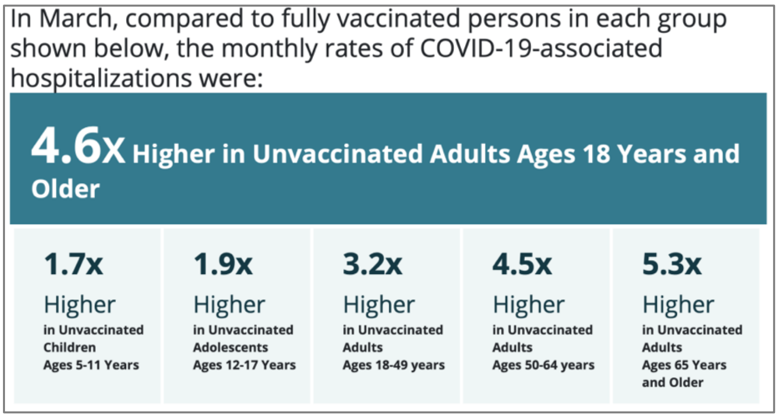

- ○ More likely
- ○ Equally likely
- ○ Less likely

*CDC-5. In your **personal opinion**, which is more risky for a young, healthy adult?*

- ○ A COVID-19 infection.
- ○ A COVID-19 vaccination.
- ○ Infection and vaccination are equally risky.
- ○ I do not know.

CDC-6. Please read the following information from [the CDC Safety of COVID-19 Vaccines] webpage about risk of vaccine side effects. If you have questions about how the vaccine's safety was tested, watch the video using link above.

Hundreds of Millions of People Have Safely Received a COVID-19 Vaccine

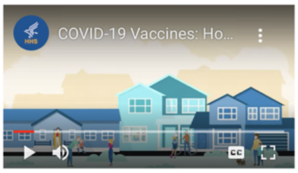

More than 576 million doses of COVID-19 vaccine had been given in the United States from December 14, 2020, through May 2, 2022. To view the current total number of COVID-19 vaccinations that have been administered in the United States, please visit the CDC COVID Data Tracker.

COVID-19 vaccines are **safe and effective**. COVID-19 vaccines were evaluated in tens of thousands of participants in clinical trials. The vaccines met the Food and Drug Administration's (FDA's) rigorous scientific standards for safety, effectiveness, and manufacturing quality needed to support emergency use authorization (EUA). Learn more about EUAs in this video.

- ○ I read the information.

CDC-7. Please read the following information from [the CDC Safety of COVID-19 Vaccines] webpages. ***According to the CDC****, which is more risky for a young, healthy adult?*

Anaphylaxis

Anaphylaxis is a severe type of allergic reaction with symptoms such as hives, difficulty breathing, low blood pressure, or significant swelling of the tongue or lips. **Anaphylaxis after COVID-19 vaccination is rare.** Learn more about COVID-19 vaccines and allergic reactions, including anaphylaxis.

Thrombosis with Thrombocytopenia Syndrome (TTS)

Thrombosis with thrombocytopenia syndrome (TTS) is a rare but serious adverse event that causes blood clots or issues with clotting. **TTS after COVID-19 vaccination is rare.** Learn more about COVID-19 vaccines and adverse events, including TTS.

Myocarditis and Pericarditis

Myocarditis is inflammation of the heart muscle, and pericarditis is inflammation of the outer lining of the heart. **Myocarditis and pericarditis after COVID-19 vaccination are rare.** Learn more about COVID-19 vaccines and adverse events, including myocarditis and pericarditis.

- ○ COVID-19 infection.
- ○ COVID-19 vaccination.
- ○ COVID-19 infection and vaccination are equally risky.
- ○ I don't know.

CDC-8. Please read the following information from [the CDC Vaccine Booster] webpage.
According to the CDC, *does the COVID-19 booster vaccination reduce the risk of hospitalization from COVID-19 infection?*

COVID-19 Vaccine Boosters

Updated May 6, 2022 Languages ▾ Print

What You Need to Know

- COVID-19 vaccine boosters can further enhance or restore protection that might have waned over time after your primary series vaccination.
- People are protected best from severe COVID-19 illness when they stay up to date with their COVID-19 vaccines, which includes a booster for many people.
- There are different COVID-19 vaccine recommendations for people who are moderately or severely immunocompromised.

- ○ Yes.
- ○ No.
- ○ I don't know.

CDC-9. Does the CDC information on vaccines seem ***trustworthy****?*

- ○ Yes, definitely.
- ○ Unsure, lean yes.
- ○ Unsure, lean no.
- ○ No, definitely not.

CDC-10. After reading the CDC's webpages, what do you believe is ***more risky*** *for you?*

- ○ COVID-19 infection.
- ○ COVID-19 vaccination.
- ○ COVID-19 infection and vaccination are equally risky.
- ○ I don't know.

CDC-11. After reading the CDC's webpage about vaccine risk, do you think a COVID-19 vaccine is more or less risky than you did before?

- ○ Less risky.
- ○ Same risky.
- ○ More risky.

CDC-12. After viewing the CDC webpages, do you intend to get a COVID-19 vaccine in the future? Or, if already vaccinated, do you intend to get a first or second booster if it is recommended?

- ○ Yes, definitely.
- ○ Unsure, lean yes.
- ○ Unsure, lean no.
- ○ No, definitely not.

6. Demographic Info

D-1. What is your age?

D-2. What best describes your gender identity?

- ○ Man.
- ○ Woman.
- ○ Non-binary.
- ○ Prefer to self-describe: ____
- ○ Prefer not to say

D-3. What is the highest degree or level of education you have completed? (R8)

- ○ Less than 8th grade.
- ○ Some high school, no diploma.
- ○ High school graduate, diploma or the equivalent.
- ○ Some college credit, no degree.
- ○ Trade/technical/vocational training.
- ○ Associates degree.
- ○ Bachelor's degree (for example: BA, BS).
- ○ Graduate degree.

D-4. Are you of Latino, Hispanic, or Spanish origin?

- ○ Yes.
- ○ No.
- ○ Prefer not to say.

D-5. How do you identify your race? Please select all that apply

- ○ American Indian or Alaska Native.
- ○ Black/African American.
- ○ Native Hawaiian or Pacific Islander.
- ○ White/Caucasian.
- ○ Asian.
- ○ Latino.
- ○ Prefer to self-describe: ____
- ○ Prefer not to say

D-6. Do you usually think of your opinions as more similar to Republicans or Democrats?

- Republican.
- Lean Republican.
- Democrat.
- Lean Democrat.
- Independent.

Sources of Cited Items:

Lazarus, J. V., Wyka, K., White, T. M., Picchio, C. A., Rabin, K., Ratzan, S. C., Parsons Leigh, J., Hu, J., & El-Mohandes, A. (2022). Revisiting COVID-19 vaccine hesitancy around the world using data from 23 countries in 2021. *Nature communications*, *13*(1), 3801.

Loomba, S., de Figueiredo, A., Piatek, S. J., de Graaf, K., & Larson, H. J. (2021). Measuring the impac t of COVID-19 vaccine misinformation on vaccination intent in the UK and USA. *Nature Huma n Behaviour*, *5*(3), 337-348.

The number of questions in coding and excel file does not precisely match this document. This table shows relationship between question numbers on participant survey and question numbers in excel files.

**Codes numbers in data**	**Survey Question numbers**
R1	V-1
R2a, R2b	D-5
R3	D-4
R4	RL-1
R5	RP-7
R6	RP-8
R7	RP-6
R8	D-3
R9	RP-4
R10	RP-2
R11	RP-1
R12	D-6
R13	D-2
R14	D-1
R15	RP-0
